# Xpg limits the expansion of haematopoietic stem and progenitor cells after ionising radiation

**DOI:** 10.1093/nar/gkw376

**Published:** 2016-05-02

**Authors:** Alush I. Avila, Anett Illing, Friedrich Becker, Lars D. Maerz, Yohei Morita, Melanie Philipp, Martin D. Burkhalter

**Affiliations:** 1 Leibniz Institute on Aging, Fritz Lipmann Institute, 07745 Jena, Germany; 2 Department of Internal Medicine I, Ulm University, 89081 Ulm, Germany; 3 Institute for Biochemistry and Molecular Biology, Ulm University, 89081 Ulm, Germany

## Abstract

Reduced capacity of genome maintenance represents a problem for any organism, potentially causing premature death, carcinogenesis, or accelerated ageing. Strikingly though, loss of certain genome stability factors can be beneficial, especially for the maintenance of tissue stem cells of the intestine and the haematopoietic system. We therefore screened for genome stability factors negatively impacting maintenance of haematopoietic stem cells (HSC) in the context of ionising radiation (IR). We found that *in vivo* knock down of Xeroderma pigmentosum, complementation group G (Xpg) causes elevation of HSC numbers after IR treatment, while numbers of haematopoietic progenitors are elevated to a lesser extent. IR rapidly induces Xpg both on mRNA and on protein level. Prevention of this induction does not influence activation of the checkpoint cascade, yet attenuates late checkpoint steps such as induction of p21 and Noxa. This causes a leaky cell cycle arrest and lower levels of apoptosis, both contributing to increased colony formation and transformation rates. Xpg thus helps to adequately induce DNA damage responses after IR, thereby keeping the expansion of damaged cells under control. This represents a new function of Xpg in the response to IR, in addition to its well-characterized role in nucleotide excision repair.

## INTRODUCTION

DNA damage poses a constant threat for the integrity of the genome and various sources generate a plethora of biochemically distinct DNA lesions (1). In order to cope with this threat elaborate mechanisms to sense and subsequently repair DNA lesions have evolved (2). Each of these pathways reverts specific kinds of damage and together they maintain genome integrity. However, if DNA lesions are not adequately addressed, cells may die or experience mutations potentially contributing to carcinogenesis. This is especially a concern for stem cells, which constantly replenish organs with newly generated mature cells (3). DNA damage can prematurely deplete stem cells, which ultimately causes insufficient organ regeneration. Moreover, generation of mutated progeny due to mutated stem cells potentially alters organ function and contributes to carcinogenesis (4).

Genome maintenance is facilitated by several groups of genes, such as repair genes (e.g. Mlh1, Brca2, Lig4 or Ercc1), and checkpoint inducers that often also function to recruit DNA damage recognition as well as repair proteins (e.g. ATM, ATR or Brca1). Mutations in genome stabilisers often have severe consequences such as embryonic lethality, early onset of cancer, or a shortened life span (5–11). Moreover, depletion of stem cells often is a hallmark of these phenotypes (12–15). Surprisingly however, the contrary can also be observed. In the context of dysfunctional telomeres, which are recognized as DNA double strand breaks (DSBs), loss of Exo1, Cdkn1a or Puma improves intestinal stem cell function and organ maintenance in mice (16–18). Similarly, also in presence of dysfunctional telomeres, knock down (KD) of Brca2 improves the capacity of murine haematopoietic stem and progenitor cells to reconstitute bone marrow after transplantation into lethally irradiated mice (19). Thus, at least some factors involved in genome maintenance negatively impact stem cell function in the presence of DNA damage such as uncapped telomeres. This prompted us to search for additional *bona fide* genome stability factors that negatively impact stem cell maintenance. To this end, we performed an *in vivo* functional genomics shRNA screen, in which we identified Xeroderma pigmentosum, complementation group G (Xpg), encoded by the gene Ercc5, as such factor. Xpg is a component of the core machinery of nucleotide excision repair (NER) (20,21). The NER machinery removes bulky adducts from the genome and recognizes these according to two different hallmarks: helix-distorting lesions in nontranscribed regions of the genome (global-genome NER) and stalled RNA polymerases II on transcribed DNA strands (transcription-coupled NER) (2). Dysfunctional global-genome NER causes Xeroderma Pigmentosum (XP), a disease accompanied with highly increased cancer susceptibility, especially in the skin (2), while defective transcription-coupled NER induces Cockayne syndrome (CS), which is characterized by severe premature ageing and lack of cancer susceptibility (2). The endonucleolytic activity of Xpg helps to release bulky lesions from genomic DNA (22,23). Mutations abolishing this activity cause XP (2). Truncation mutations of Xpg, however, cause CS in addition to XP (2). Here, we found that *in vivo* KD of Xpg elevates the number of haematopoietic stem cells (HSCs) and early haematopoietic progenitors after sub-lethal doses of ionising radiation (IR). Xpg was so far unknown to play a role in the response to IR, but is transcriptionally induced shortly after irradiation. Prevention of Xpg induction did not alter checkpoint induction on the level of p53 phosphorylation, but reduced the upregulation of DNA damage response effector genes such as p21 or Noxa. This in turn reduced cell cycle arrest and induction of apoptosis, leading to increased transformation rates after IR. Taken together, in addition to its well-characterized role concerning NER, we found Xpg to have additional functions in the response to IR.

## MATERIALS AND METHODS

### Mice and cell lines

Mice were all of the C57BL/6J background, maintained in a pathogen-free facility and fed a standard diet. All animal experiments were done in accordance with the local governments of Baden-Württemberg (35/9185.81-3/919) and Thüringen (03-006/13). Lenti-X™ 293T cells (Clontech Laboratories) and NIH-3T3 cells were maintained at 37°C and 5% CO_2_ and cultured in DMEM (Sigma) supplemented with 10% FBS (Lonza) and 1% penicillin and streptomycin (Gibco). Mouse embryonic fibroblasts were cultured in the same medium, but under low oxygen conditions (3% O_2_).

### Harvest and transplantation of haematopoietic cells

Bone marrow of 12-week-old donor mice was isolated from hind legs, front legs, hip and spine by crushing the bones in a mortar and washing the cells in sterile phosphate-buffered saline (PBS), followed by selective lysis of erythrocytes. Magnetic activated cell sorting (MACS, Miltenyi) was applied to first enrich lineage negative cells (biotin-coupled antibodies for Gr1, Ter119, B220, CD11b, CD3, CD4, CD8 (all Biolegend); streptavidin-coupled magnetic beads (Miltenyi)) or cKit-expressing cells (anti-cKit-APC antibody (Biolegend); magnetic anti-APC beads (Milteny). cKit-positive cells were further stained to allow sorting of LSK cells (lineage^−^ Sca-1^+^ cKit^+^; antibodies of eBioscience) by flow cytometry using a FACSAriaII (BD Biosciences). Haematopoietic cells were maintained in StemSpan SFEM medium (Stem Cell Technologies) supplemented with mSCF and mTPO (50 μg/ml; Peprotech) and 1% penicillin and streptomycin (Gibco). For transplantation, transduced cells were intravenously injected into lethally irradiated (12 Gy) 8-week-old wild-type mice. Recipient mice were treated during 10 days with Baytril (0.1% in drinking water; Bayer).

### shRNA libray, lentiviral production, and transduction

**Figure 1.**
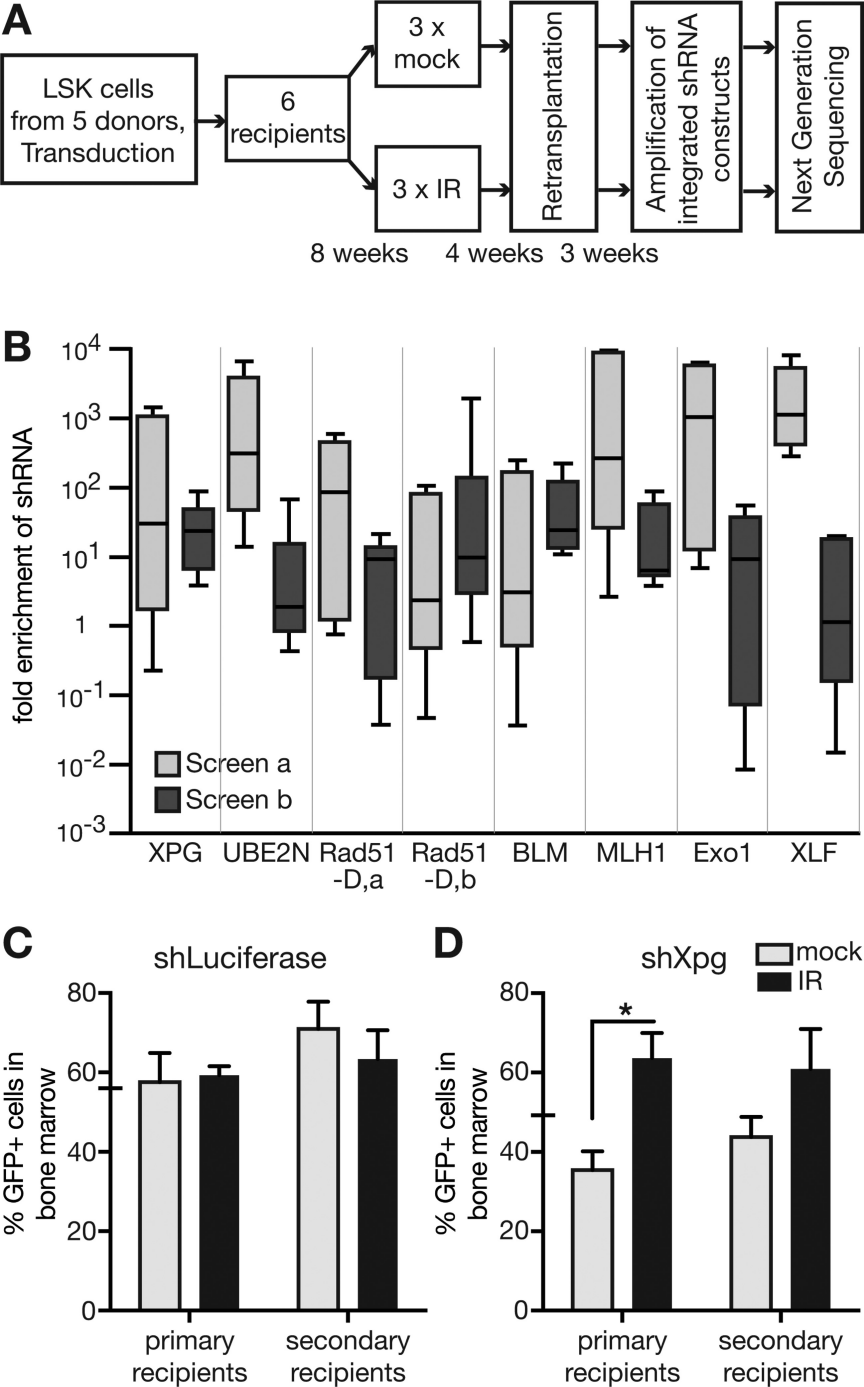
*In vivo* shRNA screen reveals role of Xpg in response to ionising radiation. (**A**) Outline of the screening procedure. (**B**) Results of two independent screens. Box and whiskers graphs summarise all possible pairwise comparisons of irradiated and mock treated mice, respectively. (**C** and **D**) To verify candidate constructs, LSK cells were transduced with individual shRNAs targeting either Luciferase as control (shLuciferase, (C)) or Xpg (shXpg-A (D)) and transplanted into lethally irradiated mice (10 mice per construct). Ticks on the Y-axis indicate the percentage of GFP-expressing cells of the transplanted cell population. After 8 weeks, 5 recipients were sub-lethally irradiated with 4 Gy, while the remaining 5 mice were left untreated. After 12 weeks bone marrow was harvested, the amount of GFP-expressing cells assessed (primary recipients), and re-transplanted into secondary recipients. 4 weeks later, the frequency of GFP-expressing bone marrow cells in secondary recipients was assessed (secondary recipients). *P < 0.05; two-tailed, unpaired Student's *t*-test.

**Figure 2.**
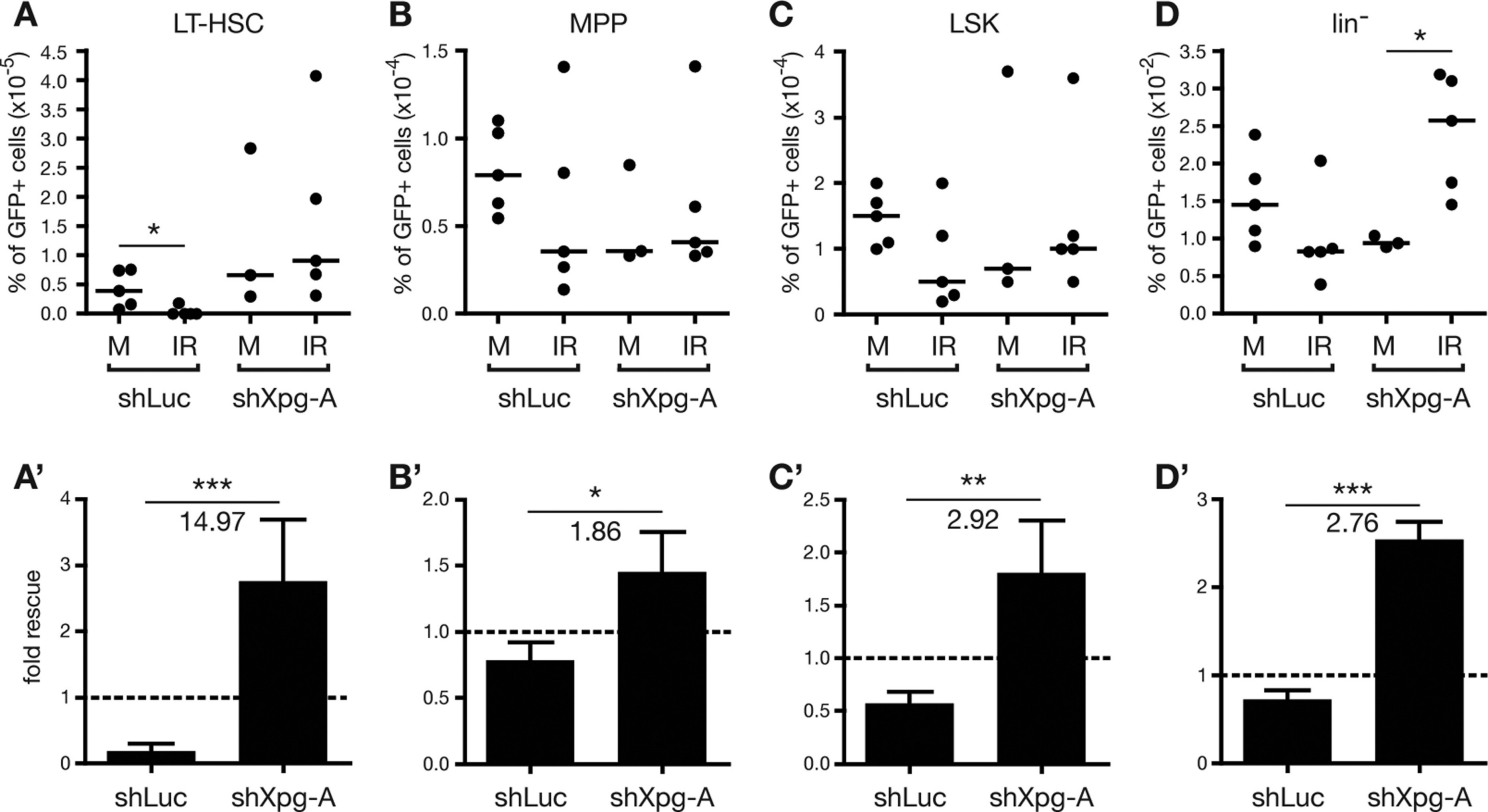
KD of Xpg allows expansion of haematopoietic stem cells after ionising radiation. Bone marrow composition of secondary recipients of cells transduced with a control shRNA (shLuc) or an shRNA against Xpg (shXpg-A) was analysed by flow cytometry (see markers in Materials and Methods section). Transplanted cells were harvested from mock treated (M) or irradiated (4 Gy; IR) donor mice. The frequency of (**A**) long-term HSCs (LT-HSC), (**B**) multipotent progenitors (MPP), (**C**) LSK cells, (**D**) lineage negative (lin^−^) cells was assessed by comparison of cell numbers to total GFP-expressing bone marrow cells. The bar indicates the median. (**A’** to **D’**) Difference between mock and IR treated groups was calculated by exhaustive combination (all possible pairwise comparisons). The number indicates the median difference. Bars represent mean and standard error of the mean. *P < 0.05, **P < 0.01, ***P < 0.005; all two-tailed, Mann–Whitney test.

shRNA constructs were in the miR30 context and picked from the Expression Arrest pSHAG-MAGIC2 retroviral shRNAmir library (Open Biosystems) and shuttled into the vector SF-LV-shRNA-EGFP (19). Additional shRNA constructs were designed using http://biodev.extra.cea.fr/DSIR/DSIR.html and http://katahdin.mssm.edu/siRNA/RNAi.cgi?type=shRNA and shuttled into the same vector. For screens, a balanced pool containing all lentiviral constructs was prepared and used for virus production. For verification experiments, individual constructs were used. Please find the sequences of shRNA constructs in the supplemental material. The CaCl_2_ method was applied to co-transfect lentiviral vectors carrying shRNA constructs, pCMVdeltaR8.91 and pMD.G into HEK293T. After harvest, viral particles were concentrated by ultracentrifugation (25000 rpm, 2.5 h, 4°C) and resuspended in sterile PBS. Lentiviral particles were used to transduce NIH-3T3 cells and haematopoietic cells, while the latter was carried out in presence of polybrene (final concentration 0.8 μg/ml; Sigma). Except for the *in vivo* experiments (Figures 1 and 2, Supplemental Figure S1), GFP-expressing cells were sorted to ensure a homogenous cell population for biochemical analysis.

### shRNA recovery and deep sequencing analysis

GFP-expressing bone marrow cells were sorted and lysed in tail lysis buffer (100 mM NaCl; 10 mM Tris–HCl, pH 8.0; 25 mM EDTA; 0.5% SDS; 0.1 mg/ml Proteinase K). After incubation at 56°C during 4 h, genomic DNA was prepared by phenol/chloroform extraction and ethanol precipitation. The integrated proviral sequences were amplified using primers that annealed to the mir30 cassette and contained the linkers needed for sequencing on an Illumina platform (for details see (24)). Next-generation sequencing was carried out on a HiSeq platform (Illumina). DNA from each animal was used as a separate template and sequenced individually using barcoded primers.

### Bone marrow analysis

Total bone marrow was extracted from tibia and femur by crushing bones in a mortar. Cells were washed in PBS and stained using following antibodies coupled to dyes: Sca-1-Pacific Blue; cKit-APC; CD34-Alexa Fluor 700; Flt3-PE; FcγR-PE; Il7R-PerCP-Cy5.5 (all eBioscience). Antigens on mature cells (Gr1, Ter119, B220, CD11b, CD3, CD4 and CD8) were labelled with antibodies coupled to biotin and streptavidin coupled to APC-Cy7 (all eBioscience). Following cell types were discriminated: lineage negative (= lin^−^); LSK (lin^−^ Sca-1^+^ cKit^+^); long-term HSCs (= LT-HSC, CD34^−^ Flt3^−^ LSK); multi-potent progenitors (= MPPs, CD34^+^ Flt3^+^ LSK); myeloid progenitors (= MP, lin^−^ cKit^+^ Sca-1^low^); megakaryocyte-erythrocyte progenitors (= MEP, lin^−^ Sca-1^low^ cKit^+^ CD34^−^ FcγR^−^); granulocyte-macrophage progenitors (= GMPs; lin^−^ Sca-1^low^ cKit^+^ CD34^+^ FcγR^+^); common myeloid progenitors (= CMPs, lin^−^ Sca-1^low^ cKit^+^ CD34^+^ FcγR^−^); and common lymphoid progenitors (= CLPs, lin^−^ Sca-1^low^ cKit^low^ Flt3^+^ Il7R^+^). Data was acquired on an LSRII (BD Biosciences) and analysis was carried out with Diva or FlowJo software. Statistical analysis was done with Prism 4 (Graphpad) software by calculating the median and applying Mann Whitney Test.

### Irradiation, apoptosis, cell cycle analysis and colony formation

Mice were subjected to full body irradiation (4 Gy) in a Gammacell 40 irradiator (Theratronics) in groups of eight animals and boxes that allow free movement of animals. Cells were irradiated with 4 or 8 Gy in a Gammacell 40 irradiator or 10 J/m^2^ UVC (VL-4.C; LTF Labortechnik) and harvested at indicated time points. Alternatively, cells were fixed and permeabilised with the BD Cytofix/Cytoperm Kit (BD Biosciences) and stained with an APC-coupled antibody specific for Annexin V (eBioscience) as well as PI. Stained cells were analysed in an LSR-Fortessa with Diva software (both BD Biosciences). For cell cycle analysis, BrdU (50 μM final concentration) was added 30 min before harvesting. Cells were fixed with ethanol and DNA denatured with 0.5% Tween20 and 4N HCl. After 30 min, pH was neutralized with 1M Tris pH 8.0 and buffer changed to phosphate-buffered saline containing 0.5% Tween20 (PBST). Samples were incubated with 5 μl of anti-BrdU antibody coupled to Alexa Fluor 647 (Biolegend), before addition of PI/RNase A solution (Cell Signaling, #4087). Cells were analysed in a FACSCalibur with Cell Quest software (both BD Biosciences). To study the ability to form colonies, transduced LSK cells were irradiated with 2 Gy or left unirradiated. Single cells were then sorted into individual wells of 96-well plates containing 80 μl of medium (see above). The number of formed colonies was assessed 7–10 days later.

### qPCR

Total RNA was extracted using the Qiagen RNeasy mini kit or the Qiazol reagent (both Qiagen) and cDNA was produced with the GoScript Reverse Transcription System (Promega) or the ProtoScript II First Strand cDNA Synthesis Kit (NEB). Expression levels were analysed in duplicates or triplicates in an ABI Prism 7300 Sequence Detection system (Applied Biosystems) or a LightCycler 480 (Roche) with Absolute QPCR ROX Mix (ABGene; Thermo Fisher) and the Universal Probe Library system (Roche). Following primers and probes were used for the individual genes (5′-3′), all intron-spanning assays: Xpg, gcgaacactgtttgaagcaa and tcttcagcaagcctttcagc, probe #82; Cdkn1a (p21), tccacagcgatatccagaca and ggacatcaccaggattggac, probe #21; Noxa, cagatgcctgggaagtcg and tgagcacactcgtccttcaa, probe #15; Hmbs, tccctgaaggatgtgcctac and aagggttttcccgtttgc, probe #79; Xpa, acgagattggaaacattgttca and cgcattcttcacagatggtg, probe #81; Xpf, gcagaaaataaggagagcgaag and atcgcttgcacagatcagc, probe #49. Expression levels were compared to Hmbs.

### Western blotting

Cells were lysed in presence of proteinase and phosphatase inhibitors (Complete mini and PhoSTOP, Roche). Protein amounts were assessed via BCA assay (Sigma). Proteins were separated in precast Novex or BOLT 4–12% Bis–Tris gradient gels (Life Technologies) and blotted onto nitrocellulose membranes. Blocking was carried out with 5% milk in TBST. Antibodies (Xpg (sc-12558, Santa Cruz Biotechnology), p53 (#2524, Cell Signalling), phospho-p53 (Ser15, #12571, Cell Signalling), p21 (F-5, Santa Cruz Biotechnology), Gapdh (ACR001PT, Acris), beta-Actin (Sigma)) were detected with secondary IR-Dye coupled secondary antibodies in a Li-Cor Odyssey SA Infrared Western blot detection system. Quantification was carried out using Li-Cor software.

### Immunofluorescence

Lin^−^ cells of 12-week old mice were isolated as described above, taken up in medium, and transduced with lentiviral particels conferring GFP expression as well as a control shRNA (shLuciferase, see above) or an shRNA against Xpg (shXpg-A, see above). After two days of culture, GFP-expressing cells were sorted in a FACSAriaIII (BD Biosciences), spotted onto Poly-L-Lysine coated diagnostic microscope slides (12-well 5.2mm numbered; Thermo Scientific) and fixed with 4% paraformaldehyde. Cells were then permeabilised with 0.5% Triton X-100 (Roth) and blocked with 2% BSA (both in 1xPBS). A primary antibody against γH2Ax (5-636, Millipore) or 53BP1 (NB100-904, Novus) was applied and incubated overnight at 4°C. Secondary antibodies specific for mouse (coupled to Alexa 594; Life Technologies) or rabbit (coupled to Cy3; Anova) were used to probe for the primary antibodies. Dapi was used to counterstain for nuclei. Cells were visualized using a Leica SP5II laser scanning confocal microscope (Leica) at 40- and 63-fold magnification and LAS AF software (Leica).

### Chromatinimmunoprecipitation

Cells were cross-linked with 1% PFA in PBS for 10 min at room temperature. Cross-linking was quenched by addition of Glycine to a final concentration of 125 mM and cells were washed twice with ice-cold PBS. Crosslinked cells were resuspended in nuclei extraction buffer (25 mM HEPES, pH 7.8; 1.5 mM MgCl2; 10 mM KCl; 0.1% NP-40; 1 mM DTT; 1x Roche cOmplete Prot. Inh.) and incubated on ice for 10 min. Nuclei were pelleted by centrifugation for 10 min at 12 000 g and resuspended in 130 μl sonication buffer (50 mM HEPES, pH 7.8; 140 mM NaCl; 1 mM EDTA; 1% Triton-X 100; 0.1% SDS; 0.1% sodium deoxycholate; 1x Roche cOmplete Prot. Inh.). Chromatin was sheared using a Covaris M220 with Snap-Cap microTUBEs (peak power 75; duty factor 9.3%; 200 cycles/burst) to a fragment size of 200–600 bp. Debris were removed by centrifugation for 10 min at 17000 g and 13 μl lysate were removed for input sample. Remaining lysates were adjusted to 500 μl with BPBS (0.5% BSA in PBS) and incubated with 5 μl Protein A/G bead mix (Dynabeads, Invitrogen) for 1 h at 4°C. Supernatant volumes equivalent to 5x10^5^ cells were removed, adjusted to 500 μl with BPBS and incubated with 4 μg antibody against Xpg (sc-12558, Santa Cruz Biotechnology) or normal goat IgG (sc-3887, Santa Cruz Biotechnology) overnight at 4°C. Chromatin/antibody complexes were absorbed to 20 μl preblocked (BPBS) Protein A/G bead mix (Dynabeads, Invitrogen) for 2 h at 4°C. Beads were washed twice with sonication buffer, twice with wash buffer I (sonication buffer; 500 mM NaCl), twice with wash buffer II (20 mM Tris, pH 8; 1mM EDTA, 250 mM LiCl; 0.5% NP-40; 0.5% sodium deoxycholate) and twice with TE buffer. Reverse-crosslinking and elution was carried out in 100 μl elution buffer (1% SDS; 100 mM NaHCO_3_; 250 mM NaCl) at 65°C for 5 h followed by Proteinase K treatment for 1 h at 45°C. MinElute columns (Qiagen) were used for DNA purification.

Purified DNA was analysed by qPCR using SYBR Green assay (iTaq Universal SYBR Green Supermix, Biorad) and 5 mM final primer concentration (p21: 5′-ccacaaccacactggctaag and 5′-ctgctacttggcaccagtttt; Control region (Cntr): 5′-agggaggctcaggaatgaac and 5′-gcaattccctcatagccagt). ΔΔCt method was used to calculate fold enrichment of a genomic locus of choice (p21 promoter region) over the ChIP specific background (Cntr.), both normalized to the signal in the input fraction. (i) ΔCt [normalized to Input] = (Ct [ChIP] − (Ct [Input] − log_2_ (Input Dilution Factor))); (ii) ΔΔCt = ΔCt [region of choice normalized to Input] − ΔCt [Cntr. region normalized to Input]]; (iii) fold enrichment = 2 (−ΔΔCt). For data visualization additional fold enrichment of Xpg over mock IgG was calculated.

### Focus formation assay

Mouse embryonic fibroblasts were plated in 6-well plates at a density of 250'000 cells per well and transduced with lentiviral particles carrying shRNA constructs for Luciferase or Xpg (shXpg-A). Cells were then irradiated (8 Gy), followed by co-transfection with plasmids carrying H-Ras (pWZL Blast H-Ras G12V E37G, Addgene #12277) and cMyc (pWZL Blast myc, Addgene #10674), respectively. In a control well, transfected cells were cultured in presence of Blasticidin to control for transfection efficiency. After 15 days, cells were washed twice with 1xPBS, fixed with ice-cold methanol and stained with 0.5% crystal violet. Plates were photographed in a Fusion-SL4 (Vilber).

### Statistics

Experiments were carried out at least three times in a biological independent fashion. If not stated otherwise, two-tailed unpaired Student's *t*-test with a confidence interval of 95% was applied. Statistical analysis was carried out with Prism 4 and 6 software (GraphPad).

## RESULTS

### *In vivo* functional genomics screen reveals Xpg to suppress expansion of haematopoietic cells after ionising radiation

We aimed to identify genome stability factors with unknown roles regarding stem cell maintenance after infliction of DNA damage. To this end, we applied an *in vivo* shRNA screening approach (outlined in Figure 1A; (19)). In order to focus our study on factors involved in genome maintenance, we generated a library containing 332 shRNA constructs targeting 145 genes with roles directly in DNA repair or associated processes (Supplemental Table S1). The chosen lentiviral construct to deliver the shRNAs simultaneously conferred GFP expression, which allowed to control for transduction efficiency (19). We produced a pool of lentiviral particles containing this shRNA library and transduced freshly isolated haematopoietic progenitor cells (LSK; see material and methods section for used markers) from 12-week-old wild type mice. Transduced cells were transplanted into lethally irradiated recipient mice. After stable bone marrow (BM) engraftment (8 weeks), half of the recipients were sub-lethally irradiated. Four weeks later, BM cells were re-transplanted into secondary recipients that also have been lethally irradiated. Finally, 4 weeks after re-transplantation, genomic DNA was extracted from FACS-sorted, GFP-expressing total BM cells harvested from the secondary recipients. This DNA was used as a template to amplify shRNA constructs stably integrated into the genome of harvested cells by taking advantage of the constant miR30 sequences (19,24). Next generation sequencing allowed the quantitative analysis of the PCR products regarding the presence of individual shRNA constructs (19,24). Samples from individual mice were kept separate at each step of the procedure. Since we were interested in genes that negatively influence stem cell functionality, we searched for shRNA constructs that were overrepresented in mice subjected to IR as compared to mock-irradiated controls. To this end, we compared shRNA read numbers obtained from irradiated individual animals to read numbers obtained from each individual control animal (all possible pairwise combinations). Furthermore, we repeated the screening procedure. On the basis of quality attributes such as fluctuation of read numbers and absolute read numbers, we obtained six candidate shRNAs (Figure 1B) and chose three of them, namely Xpg-A, Ube2n-I, and Rad51D-b for further validation. We separately produced lentiviral particles carrying either the candidate shRNA Xpg-A, Ube2n-I, Rad51D-b, or a control shRNA targeting Luciferase. In the validation experiments, we followed the same procedure as during the screening process (Figure 1A). However, we measured the content of GFP^+^ cells in the BM of primary as well as secondary recipients at the time of BM harvest to analyse the impact of conferred shRNAs. As expected, targeting Luciferase did not change the frequency of GFP^+^ cells in the bone marrow of the transplanted control mice (Figure 1C). Additionally, the observed impact of shRNAs UBE2N-I and Rad51D-b in the screens could not be recapitulated in the validation on single shRNA level. However, shRNA Xpg-A caused a significant increase of GFP^+^ cells in the BM of recipient mice only in irradiated mice (Figure 1D). Thus, KD of Xpg appears to give bone marrow cells an advantage after ionising radiation.

### Xpg reduction rescues haematopoietic stem and progenitor cells after ionising radiation

In order to study the influence of Xpg KD on different haematopoietic compartments, we analysed the BM composition of the secondary recipients in regard to haematopoietic stem and progenitor cells (HSPCs) by flow cytometry (Figure 2). Besides assessing the frequency of individual cell types, we also analysed the impact of IR on cells carrying a specific shRNA (Figure 2). In mice with the control shRNA against Luciferase (shLuc), IR treatment led to a significant reduction of GFP^+^ long-term (LT) HSCs (Figure 2A; see used markers in Materials and Methods section). Interestingly, LT-HSCs carrying the shRNA against Xpg (shXpg-A) did not show this reduction (Figure 2A). When we calculated the sensitivity to IR conferred by shXpg-A as compared to shLuc, we found a considerable rescue of LT-HSCs (Figure 2A’). Thus, KD of Xpg seems to radioprotect HSCs, the essential cells for BM maintenance and reconstitution. Analysis of frequencies of early progenitors such as multipotent progenitors (MPPs) and LSK cells revealed the same tendencies as observed on stem cell level (Figure 2B and C). Additionally, similar as to HSCs, shXpg-A caused a significant reduction of the IR sensitivity as compared to the shLuc control construct (Figure 2B’ and C’). Thus, the observed advantage conferred by Xpg KD translates from HSCs to the early progenitor compartments, although to a reduced extent. Lineage negative progenitors (Lin^−^) also showed a significant rescue in both cell number and sensitisation when carrying shXpg-A (Figure 2E and E’). This expansion is probably due to a strong shXpg-A-related effect on megakaryocyte-erythrocyte progenitors (MEPs; Supplemental Figure S1). KD of Xpg did, however, not have an effect on other more differentiated progenitors (Supplemental Figure S1).

### Reduced induction of Xpg attenuates late checkpoint responses

**Figure 3.**
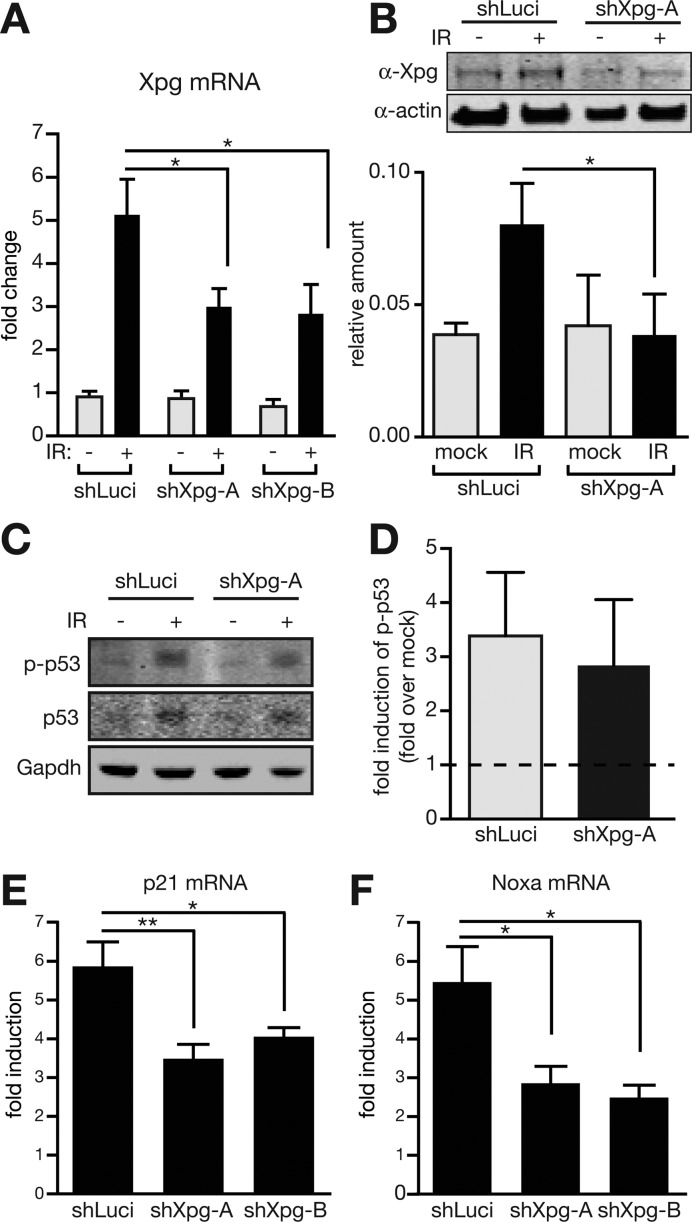
Reduced induction of Xpg after IR attenuates only late checkpoint responses. (**A**) LSK cells were freshly isolated from three mice and independently transduced with a control shRNA (shLuciferase) or either of two shRNAs for Xpg (shXpg-A or shXpg-B). Then cells were mock treated or irradiated with 4 Gy (IR). mRNA levels of Xpg were compared to expression of Hmbs 4 h after irradiation by qPCR. (**B**) Freshly isolated lineage negative cells were transduced with a control shRNA (shLuciferase) or an shRNA for Xpg (shXpg-A) and mock treated or irradiated with 4 Gy (IR). Protein levels of Xpg were compared to levels of beta-Actin 4 h after irradiation. Shown are results of three independent experiments. *P < 0.05; two-tailed, unpaired Student's *t*-test. (**C** and **D**) Freshly isolated lineage negative cells from individual mice were transduced with a control shRNA (shLuciferase) or an shRNA for Xpg (shXpg-A), sorted for GFP-expression, and mock treated or irradiated with 4 Gy (IR). Protein levels of phosphorylated p53 (Ser15), p53, and Gapdh were assessed by Western blotting 5 h after irradiation. Shown are results of three independent experiments. (**E** and **F**) Freshly isolated LSK cells from individual mice were transduced with a control shRNA (shLuciferase) or either of two shRNAs for Xpg (shXpg-A or shXpg-B) and mock treated or irradiated with 4 Gy. Induction of mRNA levels of p21 (E) or Noxa (F) was assessed 4 h later by qPCR. Fold induction of expression was calculated by comparing expression levels of irradiated to mock treated cells. Expression levels were normalized to Hmbs. *P < 0.05; **P < 0.01; two-tailed, one-way ANOVA was applied as statistical test.

In order to elucidate the mechanism how delivery of shXpg-A renders HSPCs radioresistant, we first controlled efficiency of the KD. We compared expression levels of Xpg in transduced LSK cells carrying shXpg-A or a second hairpin against Xpg, shXpg-B, to controls transfected with an shRNA against Luciferase. In unperturbed LSK cells, expression of Xpg was unaffected by either shXpg-A or shXpg-B (Figure 3A). However, when cells were analysed 5 h after irradiation with 4 Gy, we detected a considerable upregulation of Xpg mRNA in cells carrying shLuciferase. Presence of either shRNA for Xpg significantly attenuated this robust transcriptional induction (Figure 3A). We confirmed this radiation-induced upregulation of Xpg in control Lin^−^ cells on protein level by Western blotting (Figure 3B). In line with our observation on mRNA level, shXpg-A prevented Xpg upregulation by IR treatment also on protein level (Figure 3B). To test if IR causes Xpg upregulation in other cell types as well we turned to NIH-3T3 cells, widely used non-terminally differentiated murine fibroblasts. We further targeted additional NER factors with shRNAs, namely Xpa (shXpa) and Xpf (shXpf), respectively. While all shRNAs confer robust knock down, we found only Xpg to be upregulated by IR treatment (Supplemental Figure S2).

Since reduced induction of Xpg was beneficial for cell survival, we considered Xpg unlikely to be involved in repair of DNA double strand breaks (DSBs), the most relevant lesion induced by IR. Instead it appeared more likely that decreased checkpoint functions after suppressed Xpg induction allowed expansion of damaged cells, which would normally be suppressed. However, when we measured induction of DNA damage checkpoints by analysing phosphorylation of p53 at Serine 15, we did not find a difference between irradiated haematopoietic cells carrying shXpg-A or the control shRNA (Figure 3C and D). Furthermore, focus formation of early markers for DSBs, phosphorylated histone variant H2Ax (γH2Ax) as well as 53BP1, was unchanged after KD of Xpg (Supplemental Figure S3). This suggested that activation of the checkpoint cascade to the level of p53 phosphorylation is not affected in these cells. Yet, expression analysis of p21 as well as Noxa, two prominent checkpoint effectors triggering cell cycle arrest and apoptosis, respectively, revealed reduced induction of both genes after IR when cells were transduced with either of two shRNAs for Xpg as compared to a control shRNA (Figure 3E and F). We further tested this finding by making use of the stably transduced NIH-3T3 cells carrying shRNAs for Luciferase, Xpg, or Xpf (Supplemental Figure S3). Similar as to our observations in primary haematopoietic cells, Western blot analysis of NIH-3T3 cells did not reveal a difference in activation of p53 after IR treatment for neither Xpg nor Xpf KD cells (Supplemental Figure S4). However, both shRNAs prevented proper p21 induction after IR on both protein as well as mRNA level (Supplemental Figures S4 and S5). This suggests that although activation of the checkpoint cascade is unperturbed, adequate exertion of late checkpoint steps are attenuated due to Xpg or Xpf KD. Since checkpoints are not exclusively triggered by DSBs, we also induced DNA lesions by UV-C irradiation. Interestingly, while KD of Xpa, Xpf or Xpg reduces induction of p21 after IR, UV-C shows the inverse effect and elevates induction of p21 in absence of these NER factors (Supplemental Figure S5).

### Xpg KD affects cell cycle arrest and apoptosis induction after IR

**Figure 4.**
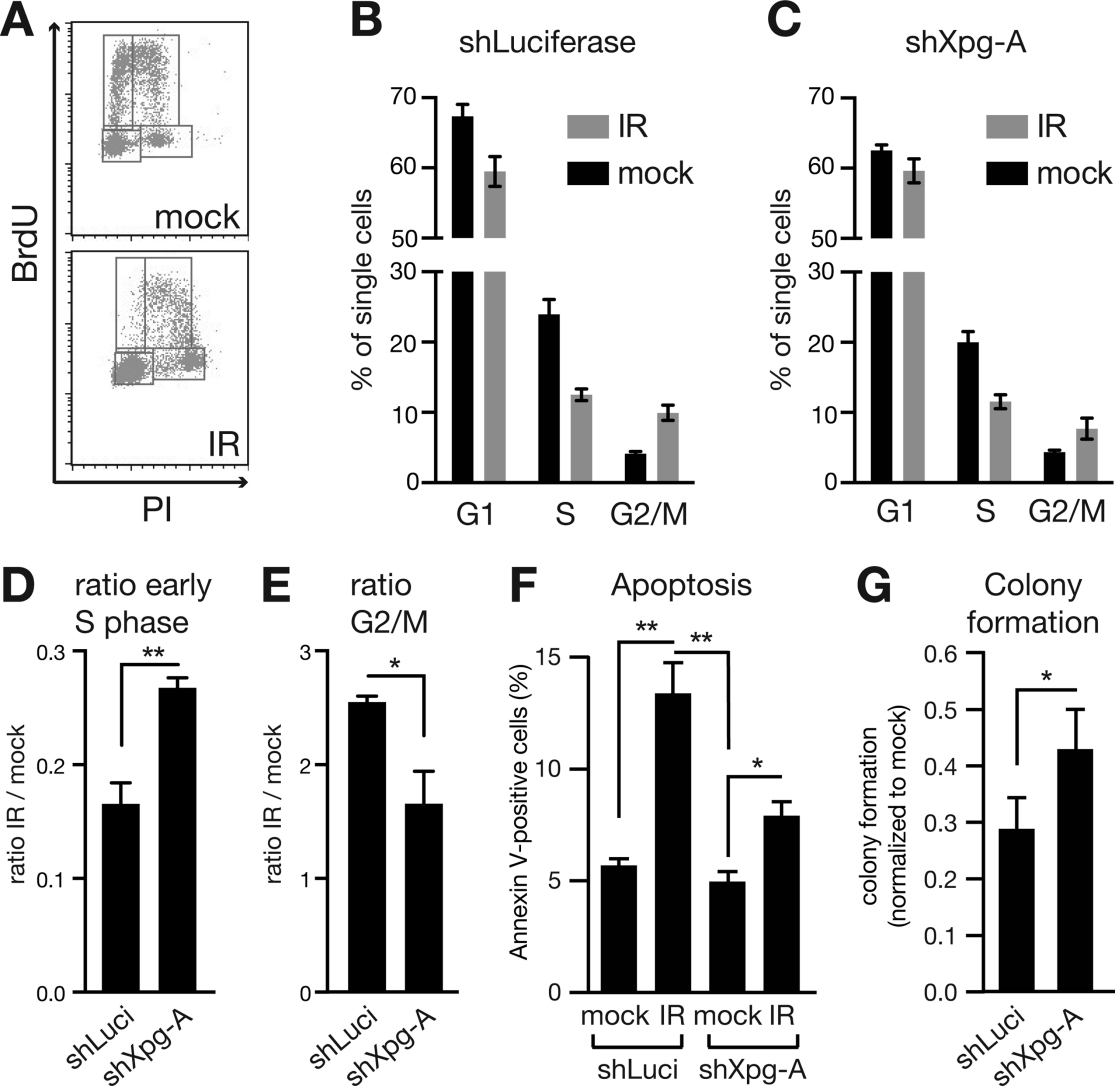
Xpg KD diminishes cell cycle arrest and apoptosis after IR. Lin- cells of individual mice were transduced with shRNAs specific for Luciferase (shLuci) or Xpg (shXpg-A) and mock treated or irradiated with 4 Gy (IR). The cell cycle status was assessed via BrdU-incorporation assay 5 h after irradiation. (**A**) Typical FACS profiles of mock treated and irradiated cells with indication of gates. (**B** and **C**) Bars represent the percentage of cells in G1, S and G2/M phases, respectively for control cells (**B**) and cells carrying shXpg-A (**C**). Comparison of percentages between related mock treated and irradiated samples revealed an increase of early S phase (**D**) and a reduction of G2/M phase (**E**) in Xpg KD cells. *P < 0.05; two-tailed, unpaired Student's *t*-test. (**F** and **G**) Freshly isolated LSK cells were transduced with a control shRNA (shLuciferase) or shXpg-A and mock treated or irradiated with 4 Gy (IR). (F) Induction of apoptosis was measured by Annexin V detection 5 h after irradiation. Two-tailed, one-way ANOVA was applied for statistics. (G) The ability to form colonies was compared to untreated cells. Two-tailed, unpaired Student's t*-*test was applied. Shown are mean and standard error of the mean of three independent experiments. *P < 0.05, **P < 0.01.

Is reduced induction of p21 or Noxa due to Xpg KD of relevance for irradiated cells? To test this question, we analysed cell cycle profiles of Lin^−^ cells transduced with shLuciferase or shXpg-A after optional IR treatment. We found that under unperturbed conditions Xpg KD cells show slightly less cells undergoing S phase as compared to control cells (Figure 4A–C). Additionally, IR substantially reduced early S phase in cells carrying either shRNA. However, this reduction was attenuated in Xpg KD cells (Figure 4D). Moreover, IR caused increased numbers of cells in G2/M, what was diminished by Xpg KD (Figure 4E). A similar increase in S phase cells and decrease in G2/M phase cells we observed after irradiation of Xpf and Xpg KD NIH-3T3 cells (Supplemental Figure S4G and H). Thus, cell cycle arrest seems to be reduced, if Xpg cannot be properly induced. To see if KD of Xpg similarly affects apoptosis, we measured Annexin V-positive cells after IR. This analysis revealed decreased induction of apoptosis in Xpg KD cells as compared to controls (Figure 4F). The combination of leaky cell cycle arrest and cells evading apoptosis could explain the improved ability to form colonies after IR of cells carrying shXpg-A (Figure 4G).

### IR recruits Xpg to p21 promoter

**Figure 5.**
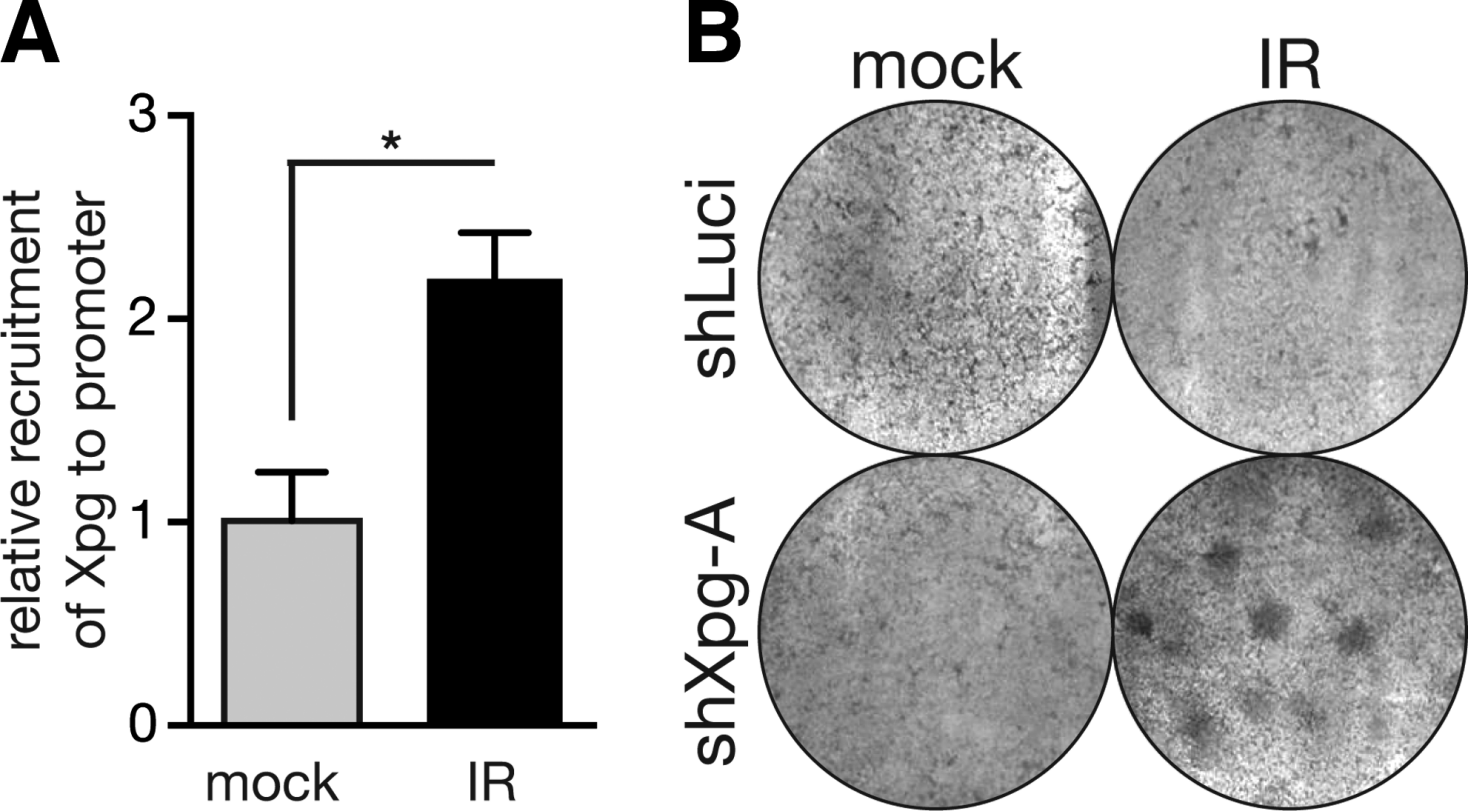
IR recruits Xpg to p21 promoter to prevent transformation. (**A**) Freshly isolated lineage negative cells were mock treated or irradiated with 4 Gy (IR), followed by crosslinking of protein/DNA complexes. Subsequent chromatin immunoprecipitation analysis revealed IR-dependent recruitment of Xpg to the promoter region of the Cdnkn1a (p21) gene as compared to mock treated cells. Shown are mean and standard error of mean of three independent experiments. *P < 0.05; two-tailed, unpaired Student's *t*-test was applied. (**B**) In focus formation assay (Ras/Myc transformation assay) of mouse embryonic fibroblasts transduced with either a control shRNA against Luciferase (shLuci) or an shRNA targeting Xpg (shXpg-A), patches of transformed cells were only observed in plates with Xpg KD cells additionally subjected to IR.

Xpg has been identified previously to be important for adequate transcriptional induction of activated target genes of nuclear receptors (25,26). Xpg forms a stable complex with TFIIH (25) and together with Xpf is recruited to promoters and terminators of nuclear receptor target genes upon stimulation such as retinoic acid receptor β2 (Rarβ2). The mediator complex probably also plays a role in this recruitment (27). At these sites, the two endonucleases are proposed to induce nicks, what triggers DNA demethylation and subsequent recruitment of CCCTC binding factor (CTCF) chromatin organizer (26). CTCF promotes looping between promoter and terminator of the Rarβ2 gene, which induces robust transcriptional induction of target genes. We wondered if Xpg could play a similar role concerning the adequate induction of checkpoint target genes. To this end, we performed chromatin immunoprecipitation assays (ChIP) of mock treated and irradiated Lin- cells. We detected an IR-dependent recruitment of Xpg to the promoter region of the Cdkn1a gene, which encodes p21 (Figure 5A). Thus, similar as to target genes of other signalling cascades, Xpg seems to be involved in proper induction of checkpoint effector genes.

A key function of the DNA damage checkpoint cascade is to provide a time window for DNA repair and repress expansion of damaged cells. Failure of this function can allow elevated levels of transformation leading to the onset of cancer. To estimate whether KD of Xpg has an influence on the transformation rate of cells after IR, we performed focus formation assays. IR treatment or Xpg KD alone did not cause formation of foci of transformed cells. However, we observed a considerable increase of cell foci after IR treatment of Xpg KD cells (Figure 5B). This suggests that inadequate induction of checkpoint effector genes due to loss of Xpg puts cells at elevated risk to become transformed contributing to carcinogenesis.

## DISCUSSION

In this study, we searched for genome stability factors that have a negative impact on HSCs and the haematopoietic system after induction of DNA damage. The most robust hit we obtained was Xpg, a key component of the core NER machinery (28). This pathway reverts bulky adducts, which can be caused by a wide variety of sources (29). Reduced NER function often presents with hypersensitivity of the skin to ultraviolet (UV) light due to the skin's pronounced exposure to sunlight. In contrast, the haematopoietic system probably is not as prominently targeted by helix-distorting DNA lesions and possibly therefore not as obviously affected by NER defects early in life. Nevertheless, dysfunctional NER also was found to have varying consequences for the haematopoietic system. A leukaemia cell line has been established from mice bearing a mutation in the gene encoding Xpg (30) and polymorphisms in some NER factors have been reported to be leukaemia risk factors (31,32). Moreover, bone marrow failure (33) and cases of leukaemia at later stages in life (34) have been reported in XP patients implicating NER factors in haematopoietic degeneration. This correlates with results obtained in animal models of accelerated depletion of HCSs and thus segmental premature ageing of the haematopoietic system after loss of Ercc1 or Xpd (13,14). Additionally, detoxification problems of endogenous aldehydes, which induce bulky adducts and interstrand crosslinks similarly cause premature failure of HSCs (35). These observations could be explained by the exquisite sensitivity to interstrand crosslinks, the repair of which is promoted by Ercc1 and Xpd, and partly by Xpg (36).

Here, we report a previously underappreciated role of Xpg in the response to IR. IR rapidly induces Xpg both on the mRNA as well as protein level (Figure 3). Yet, Xpg is not likely to be involved in the repair of lesions caused by IR, since primary fibroblasts from patients carrying mutations in the Ercc5 gene only show limited sensitivity to IR (37). Moreover, Xpg KD rather causes an expansion of cell numbers, both *in vivo* (Figures 1 and 2) as well as *in vitro* (Figure 4). This indicates that Xpg is limiting the expansion potential of haematopoietic cells after IR.

Our results indeed suggest that Xpg is involved in proper induction of checkpoint responses. Checkpoint responses are highly complex cascades and thus can be altered or interrupted at different levels (38). Since activation of upstream factors as measured by phosphorylation of p53 was not altered, Xpg seems to be needed at later steps of the checkpoint cascade. In line with this, induction of p53 target genes, which are among the latest effectors of the checkpoint response, was insufficient after KD of Xpg. How attenuation of Xpg induction exactly exerts this effect remains to be investigated. One piece of the puzzle is our observation that IR causes recruitment to the p21 promoter, where Xpg potentially could induce nicks via its endonucleolytic activity. This is similar to studies, which have reported Xpg at promoters of nuclear receptor target genes (25,26). These studies furthermore revealed a role for Xpa and Xpf in this process (26), what we here could recapitulate: in our hands KD of Xpf and Xpa caused a similar attenuation of p21 induction as Xpg KD. Additional mechanisms may play into our observation, too, as Xpg potentially also enhances transcriptional elongation of p21 mRNA (39). Moreover, p53 target genes such as Puma are induced both by glucocorticoid treatment and IR (40). It is thus conceivable that Xpg could play a similar role at the promoters of these genes as compared to the Rarβ2 gene.

Important to note is, however, that although p21 induction is reduced after IR in Xpg, Xpa or Xpf KD cells, UV-C even enhances p21 induction in these cells. This could well be due to unrepaired bulky lesions, since NER factors are targeted for KD. This would result in prolonged upstream checkpoint signalling. Additionally, this suggests alternative mechanisms to boost p21 transcription levels in the case of bulky DNA adducts as compared to DSBs. Such differences could also explain, why Xpg KD increases cell number after IR, while the Xpd knock out mouse shows premature bone marrow failure (14).

Most intriguing, however, is that mainly HSCs and to a lesser extent early progenitors are radioprotected by KD of Xpg as compared to more differentiated haematopoietic progenitors. HSCs thus possibly trigger checkpoint responses via different mechanisms than more differentiated cells would do. Knowledge about such differences could thus provide potential targets for stem cell specific therapies. Further studies to this subject would thus be of great interest, also to provide additional bases for therapeutic strategy developments given that the elevated transformation rate as in the case of Xpg KD could be prevented. Not least because short-term targeting of NER factors specifically in the haematopoietic system could have the advantage of relatively low side effects as judged by the low impact of NER mutations on the haematopoietic system.

## Supplementary Material

SUPPLEMENTARY DATA
