# Supplementary material for: Xpg limits the expansion of haematopoietic stem and progenitor cells after ionising radiation
Source: Nucleic Acids Res. 2016 May 2;44(13):6252–61. doi: 10.1093/nar/gkw376 (PMC5291257; doi:10.1093/nar/gkw376)
Supplement: SUPPLEMENTARY DATA [file supp_44_13_6252__index.html]

Xpg limits the expansion of haematopoietic stem and progenitor cells after ionising radiation — Xpg limits the expansion of haematopoietic stem and progenitor cells after ionising radiation — SUPPLEMENTARY DATA 

# Xpg limits the expansion of haematopoietic stem and progenitor cells after ionising radiation

## SUPPLEMENTARY DATA

- SUPPLEMENTARY DATA
