# Supplementary material for: Xpg limits the expansion of haematopoietic stem and progenitor cells after ionising radiation
Source: Nucleic Acids Res. 2016 May 2;44(13):6252–61. doi: 10.1093/nar/gkw376 (PMC5291257; doi:10.1093/nar/gkw376)
Supplement: SUPPLEMENTARY DATA [file supp_gkw376_nar-03028-d-2015-File007.pdf]

Avila et al., Supplemental Material

Supplemental Figure 1

KD of Xpg allows megakaryocyte/erythrocyte progenitors to expand after ionising radiation. Bone marrow composition of secondary recipients of cells transduced with a control shRNA (shLuc) or an shRNA against Xpg (shXpg-A) was analysed by flow cytometry (see markers in materials and methods section). Transplanted cells were from mock (M) or IR treated (4 Gy; IR) donor mice. The frequency of (A) myeloid progenitors (MP), (B) megakaryocyte-erythrocyte progenitors (MEP), (C) common myeloid progenitors (CMP), (D) granulocyte-macrophage progenitors (GMP), and (E) common lymphoid progenitors (CLP) was assessed by comparison of cell numbers to total GFP-expressing cells. The bar indicates the median. (A' to E') Difference between mock and IR treated groups was calculated by exhaustive combination (all possible pairwise comparisons). The number indicates the median difference. Bars represent mean and standard error of the mean. *p<0.05, **p<0.01, ***p<0.005; all two-tailed, Mann-Whitney test.

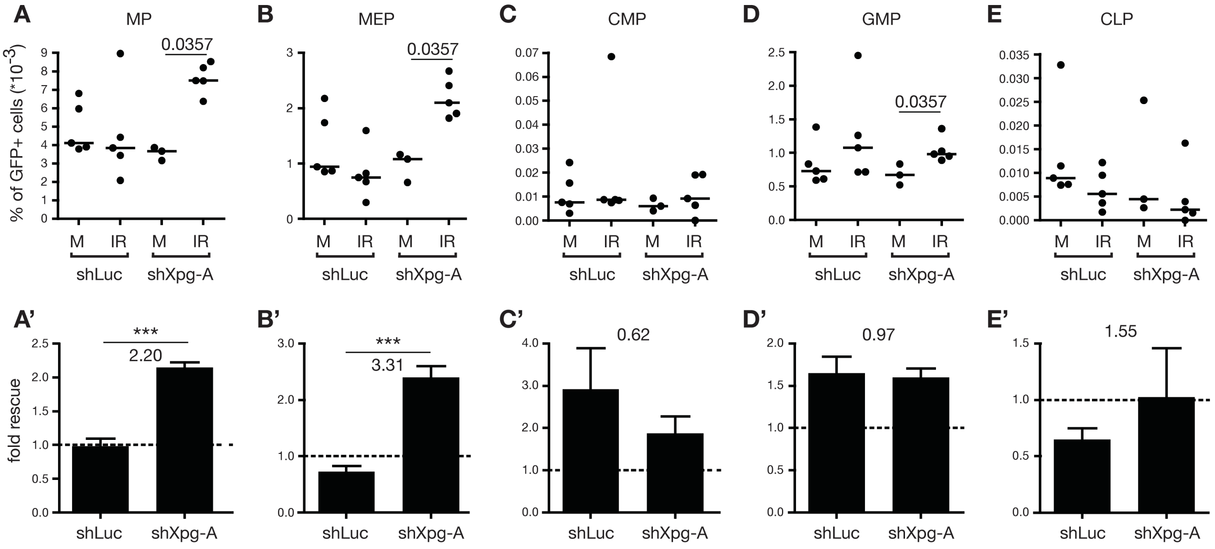

Supplemental Figure 2

Ionising radiation upregulates Xpg. NIH-3T3 cells were transduced with lentiviral particles conferring an shRNA specific for Luciferase as a control, Xpa (shXpa, for sequence see Supplemental Table 1), Xpf (shXpf, construct Xpf-II in Supplemental Table 1), or Xpg (shXpg-A). Cells were either mock treated or irradiated with 4 Gy. 4 hours later total RNA was harvested to analyse expression levels of Xpg (A), Xpa (B), or Xpf (C). All shRNAs efficiently knock down their target. Only Xpg is significantly upregulated by IR. Shown are the results of three independent experiments. *p<0.05, **p<0.01, ****p<0.001; two-tailed one-way ANOVA.

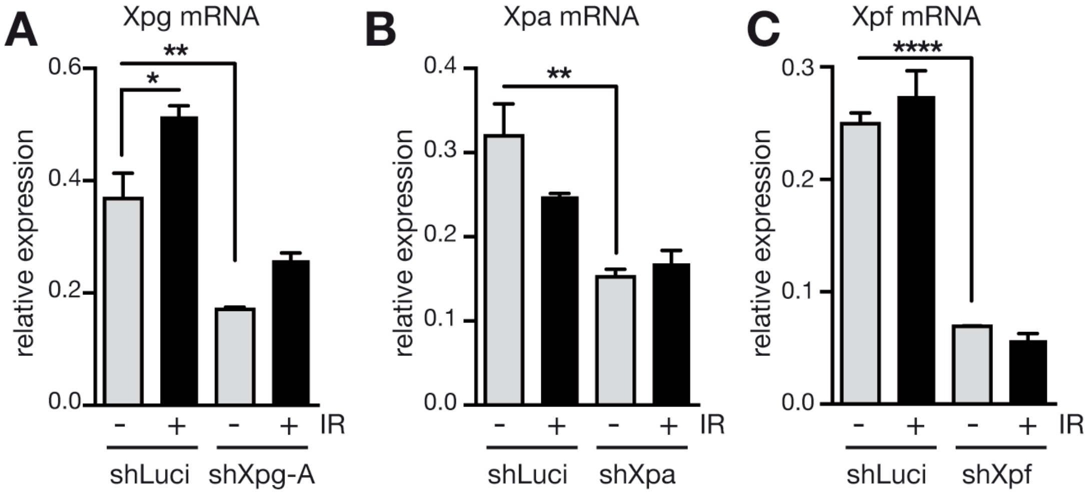

Supplemental Figure 3

KD of Xpg does not affect the early response to DNA double strand breaks. Freshly isolated lineage negative cells were transduced with a control shRNA (shLuciferase) or an shRNA for Xpg (shXpg-A) and mock treated or irradiated with 4 Gy (IR), fixed 4 hours after irradiation, and stained for Dapi and an antibody specific for phosphorylated H2Ax (γH2Ax, (A and B)) or 53BP1 (C and D). (A) Representative examples of cells probed for γH2Ax. (B) Quantification of cells probed for γH2Ax. (C) Representative examples of cells probed for 53BP1. (D) Quantification of cells probed for 53BP1. Shown are cells carrying shLuciferase. Quantification of three independent experiments. Two-tailed, one-way ANOVA was applied as statistical test. ***p<0.005

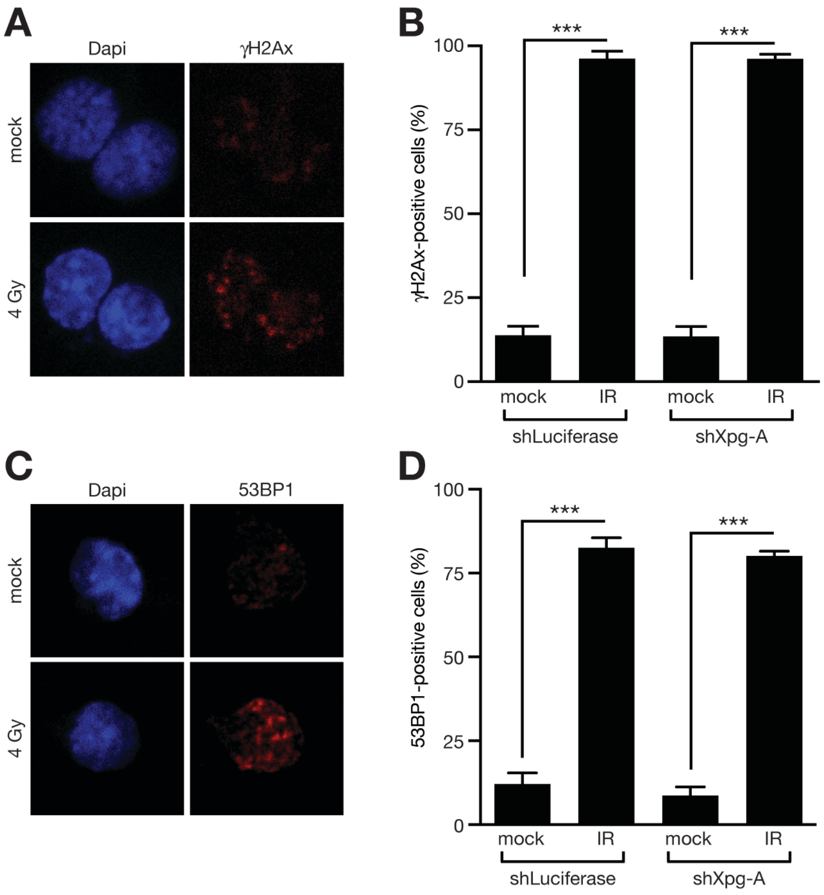

Supplemental Figure 4

Knock down of Xpg or Xpf in NIH-3T3 cells reduces late checkpoint responses while activation of p53 is unaffected. NIH-3T3 cells transduced with lentiviral particles carrying an shRNA targeting Luciferase as control (shLuci), Xpg (shXpg-A) or Xpf (shXpf) were mock treated or irradiated with 4 Gy. 5 hours post-irradiation, proteins or RNA were harvested for analysis. Western blotting revealed that KD of neither Xpg (A and C) nor Xpf (B and D) affected checkpoint activation after IR as measured by phosphorylation of p53 as compared to the control. However, KD of either Xpg (A and E) or Xpf (B and F) attenuated induction of p21 after IR as compared to the controls. Unpaired, two-tailed Student's T test was applied (*$p<0.05$, ***$p<0.005$). (G and H) Reduced p21 induction was reflected in altered cell cycle profiles of cells with lower levels of Xpg or Xpf 24 hours after irradiation. BrdU-incorporation analysis revealed elevated levels of S phase cells (G), with simultaneous reduction of G2/M cells as compared to the control after KD of Xpg or Xpf. Difference between mock and IR treated groups was calculated by exhaustive combination (all possible pairwise comparisons). Bars represent mean and standard error of the mean. **$p<0.01$, ***$p<0.005$, ****$p<0.001$; two-tailed Kruska-Wallis test.

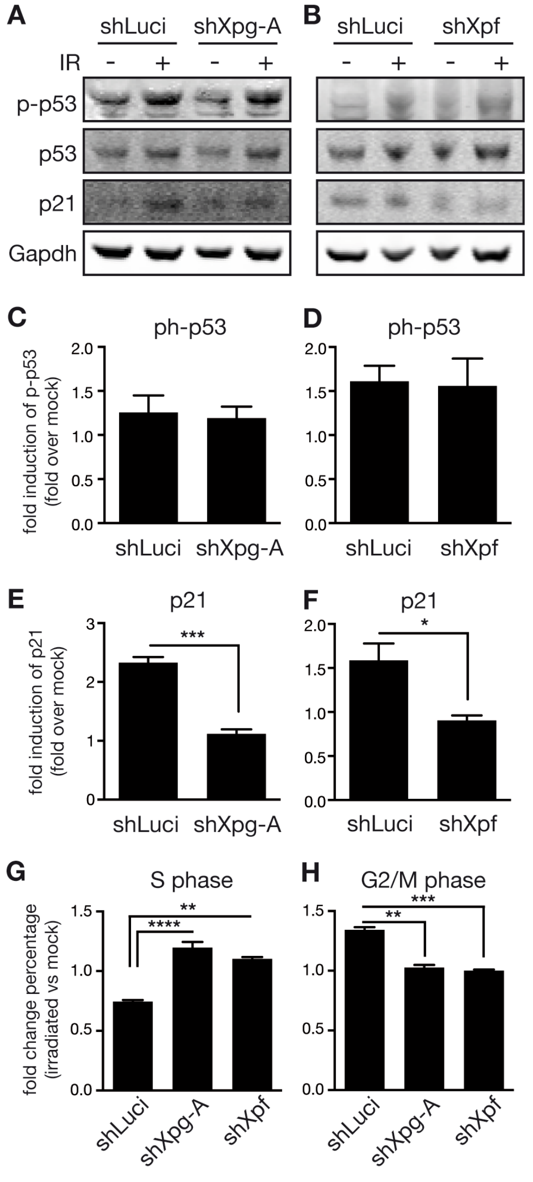

Supplemental Figure 5

Reduction of Xpa, Xpf or Xpg inversely affects p21 induction after IR and UVC treatment. NIH-3T3 cells stably expressing shRNAs targeting Luciferase (shLuci) as control, Xpa (shXpa), Xpf (shXpf) or Xpg (shXpg) were irradiated with 8 Gy (A) or 10 J/m^2 UVC (B). RNA was harvested 5 hours after irradiation and induction of p21 mRNA assessed. While KD of Xpa, Xpf or Xpg attenuates p21 induction after IR (A), reduced expression of these NER factors causes increased induction of p21 after UVC treatment (B). Bars represent mean and standard error of the mean. *p<0.05; two-tailed, one-way ANOVA.

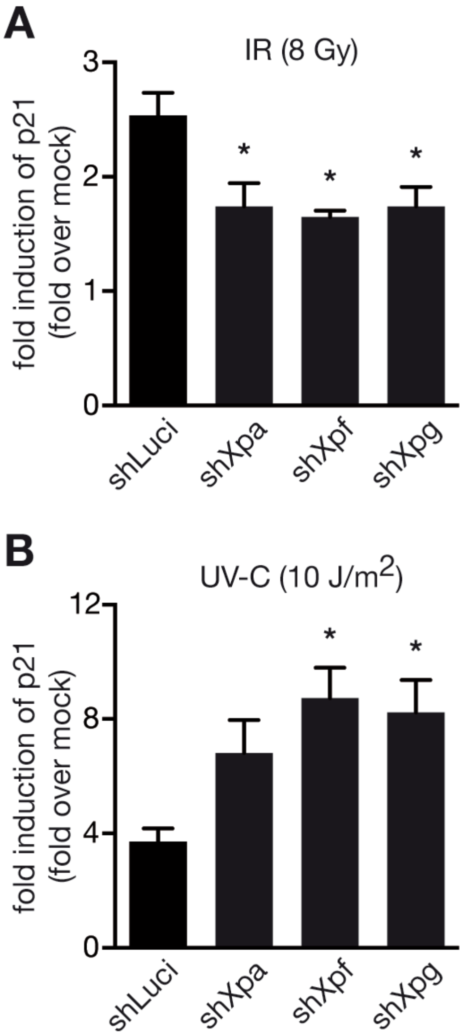

Supplemental Table 1
shRNAs represented in the lentiviral library used for screening as well as additional constructs used as controls and verification experiments. shRNAs were imbedded in the miR30 context (for details see (19,24)). Indicated are names of constructs, guide sequence, target genes and representation in library.

Name of construct	Sequence	target	included in screen
Luciferase	CCGCCTGAAGTCTCTGATTAA	M15077	no
APEX1-B	CCGAAAGTTTCTAAAGGACTT	NM_009687.1	yes
APEX1-C	CCGGGTGATTGTGGCTGAATT	NM_009687.1	yes
APTX	GCCGGCAGAGTGACTGTTAAA	NM_001025444, NM_025545, XM_001477288	yes
Areg	CCGGCTATATTATAGATGATT	NM_009704	yes
Artemis I	CCAGTGAATGTGTATCCAAAT	NM_001110214, NM_146114, NM_175683	yes
Artemis II	CCCAGTGAATGTGTATCCAAA	NM_001110214, NM_146114, NM_175683	yes
ATR	CCAGACCAGATCATTCATTAT	NM_001184, XM_001131387, XM_941191	yes
ATRIP	GCAGAGAAGATATTCCAGTTA	NM_001106859, NM_172774	yes
Axl I	CCAGATGGAGTTTCTAAGCTA	NM_009465	yes
Axl II	GGTCATCTTGCCTTTCATGAA	NM_009465	yes
BARD1-A	TAGCATCAAACTACTTGTTAA	NM_007525.3	yes
BARD1-B	AAGGAAGAAGAATTCAATAAA	NM_007525.3	yes
BARD1-C	TTGGTTTATGTTAGCTTTTAA	NM_007525.3	yes
BC030336	CCTAATATTTCCAATAGGATT	BC030336, XM_133786	yes
BCCIP I	CCTGCTGATTAGTAAGACATT	NM_025392	yes
BCCIP II	GCTGCTTCTACCTGCTGATTA	NM_025392	yes
BLM I	GGCATCCTAATAAAGAGTTAA	NM_001042527, NM_007550	yes
BLM II	GCAAATATTTACCTCCAATTA	NM_001042527, NM_007550	yes
BRCA1 I	CCACAGAGCGTCTAGGAAATT	NM_009764	yes
BRCA1 II	CCACAAAGTGTGACCACATAT	NM_009764	yes
BRCA1 III	CCACCGATGTTCCTTGGATAA	NM_009764	yes
BRCA1 IV	GCTTTGTCCATCATCTCATTA	NM_009764	yes
BRIP1 I	GCCTTCAGTCAGAAGTCATAA	NM_178309	yes
BRIP1 II	GCACAGCTTGCGATGATGAAT	NM_178309	yes
BTBD12	CCAAAGTGCCTATAACTCCAA	NM_177472	yes
c-ABL I	GGAACCACCATTCTACATAAT	NM_001112703, NM_009594	yes
c-ABL II	CCGGCCCTCCTTTGCTGAAA	NM_001112703, NM_009594	yes
c-ABL III	GCTCTTATAAATGACATGTAA	NM_001112703, NM_009594	yes
CDK9	CCAGCCCAACCGATACACCAA	NM_001007743, NM_130860	yes
CHD4 I	CGCTTCTACCGCTATGGAATA	NM_145979	yes
CHD4 II	GCGGACACAGTTATTATATAT	NM_145979	yes
CHD4 III	GGGCCACGTTCACTATTTAAT	NM_145979	yes
CHD4 IV	GCCTGCGGAATGATAAAGATA	NM_145979	yes
CHD4 V	GGAGTCTCTGCTTCGGATAAA	NM_145979	yes
CHEK1	GCCACGAGAATGTAGTGAAAT	NM_007691	yes
CLSPN I	GGCAGCAAAGGACTCATCTAA	NM_175554	yes
CLSPN II	GCGCCAAGCATTATTTAAATT	NM_175554	yes

DCLRE1A I	GGACACACTCTAACAACATAA	NM_001106201, NM_018831	yes
DCLRE1A II	CGAGTGAAGGCAGTACACTAA	NM_001106201, NM_018831	yes
DCLRE1B I	GCTCACATTAGCAGAGCTGTT	NM_001025312, NM_133865	yes
DCLRE1B II	GGATACTTTGGAACAATTCTA	NM_001025312, NM_133865	yes
Dna2	CCAGAAGCCATGCTGTCCAAA	NM_177372	yes
EGFR I	CCATCCTCTGCAATATGGATA	NM_007912, NM_207655	yes
EGFR II	CCCGTGGTTGAGAAGCTAGAA	NM_007912, NM_207655	yes
EME1 I	CCACTCAGACATGGATTTAAT	NM_133807, NM_177752	yes
ERCC1	CCAGCCGACCTCCTTATGGAA	NM_007948	yes
ERCC1-A	CTGGTTCAAGTGGATGTGAAA	NM_007948	yes
ERCC1-B	TAGGCCCAGTGTCACAATAAA	NM_007948	yes
ERCC6 I	CGAGATCAAGACCTACAAATA	AK028576	yes
ERCC6 II	GGACTTGTCTCAGTAGACTAA	AK028576	yes
ERCC6 III	GGGACCTAACAGTTGTTTAAT	AK028576	yes
ERCC6 IV	CCAGATACTTTGGAGTGAAAT	AK028576	yes
EXO1	GGGCATTATCTCAGGATGAAT	NM_012012	yes
EXO1-A	CTCGAAGAGAGAGACGACAAA	NM_012012	yes
EXO1-B	CAGGAAGTCTTGTGTGTTGAA	NM_012012	yes
FAM19A2	GGAAAGCTTCTGATAATTATT	NM_182807	yes
FANCA-A	GGAAAGCTTCTGATAATTATT	NM_016925.3	yes
FANCA-B	CTGGAAGAGCTCAGAGCTTTA	NM_016925.3	yes
FANCA-C	AAGTTTCAGTTCGTTGTGTTA	NM_016925.3	yes
FANCB I	GCAGCTATGCGGAGATGACTT	NM_175027	yes
FANCB II	GGTGGATATAACTGTAGAATA	NM_175027	yes
FANCB III	GCACCTTAATATGACAGTAAA	NM_175027	yes
FANCB IV	GGATACTGAAGCACCTTAATA	NM_175027	yes
FANCC	GCATTCAGATTCGACATGAAA	NM_001042673, NM_007985, NM_012557	yes
FANCD1 I	CCTTAGAAAGCCAATACCAAA	NM_001081001, NM_009765	yes
FANCD1 II	CGTCAGTTTAACAAAGATTTA	NM_001081001, NM_009765	yes
FANCD2 I	CCTGCTTATCCCAAAGTAATA	AK019136, NM_001033244	yes
FANCD2 II	CCAGGCACACCTTTGTCATAT	AK019136, NM_001033244	yes
FANCD2 III	GCTCTTTCAGGCCTTGAGGAA	AK019136, NM_001033244	yes
FANCD2 IV	GCATCTTAGATTATTTGGAAA	AK019136, NM_001033244	yes
FANCE-A	TTGACCAGAAGCTGTCAGTTA	NM_001163819.1	yes
FANCE-B	ATGGAGTTGCTATGCAGCGAA	NM_001163819.1	yes
FANCE-C	CAGGCTGCAGCTGCTGCTGAA	NM_001163819.1	yes
FANCF I	GCAAACCACTTTAGGAATAAT	AK042688	yes
FANCF II	GGGAAAGAGCTGAAATCATTA	AK042688	yes
FANCF III	GCAAACCACTTTAGGAATAAT	AK042688	yes
FANCG I	GGCCAGGATACCAAAGCCTTA	NM_004629, NM_053081, XM_001069391	yes
FANCG II	CCAGGCCTGGTCACAACTAAA	NM_004629, NM_053081, XM_001069391	yes
FANCG III	GCCGGGTCTCTTCCACTGTAT	NM_004629, NM_053081, XM_001069391	yes
FANCG IV	GGATCTGCTACTACTGCTAAA	NM_004629, NM_053081, XM_001069391	yes
FANCI	CCTCAGTTTGTGCAGATGTTA	NM_001113378, NM_018193, NM_145946	yes
FANCL-A	AAGCAAGAGTTGTGTGTACAA	NM_025923.2	yes
FANCL-B	CAGCTCAAGAAGGCAAGATTA	NM_025923.2	yes
FANCL-C	TTCAGAGAAAATACAAAGCAA	NM_025923.2	yes

FANCM I	GCTGTTAACAGGAAGGAGATA	NM_178912	yes
FANCM II	CCACGAATGGTTCCTGATAAA	NM_178912	yes
FANCM III	GCCAATCAAGTGAAGAAGAAA	NM_178912	yes
FANCM IV	GGTCTCAGTCTGAAATGTTAA	NM_178912	yes
FANCM V	GCAGAGCATGTTTCAAAGGAT	NM_178912	yes
FANCM VI	GCTGAAGAGATCTATAAATAT	NM_178912	yes
FEN1-A	CAGGAAAGATGTCAGGCTCAA	NM_007999.3	yes
FEN1-B	CCCGTGCTAATGCGACACTTA	NM_007999.3	yes
FEN1-C	CAGGCTCACTCTCCTCAGCTA	NM_007999.3	yes
Gabra6	GGGAGCTATGCTTATCCTAAA	NM_001099641, NM_008068	yes
GEN1 I	GGCCATGTGTGCTTACCTTAA	NM_001106717, NM_177331	yes
GEN1 II	GCGAATACTTCCTTACCTTAT	NM_001106717, NM_177331	yes
Grcc10	GGTTATTAAAGCCTATGGCTT	NM_013535	yes
GRK5 I	CCAGTTGTAACCACCGAATAA	NM_018869	yes
GRK5 II	CCTCCAAGTTTCTCAAAGAAA	NM_018869	yes
H2AFX-A	CCGCAACGACGAGGAGCTCAA	NM_010436.2	yes
H2AFX-B	AGCGACTCAACTACAACCCAA	NM_010436.2	yes
H2AFX-C	CCTGTGGACAAGAGTTCTATA	NM_010436.2	yes
Hdac2	GCTGCTAAATTATGGTTTATA	NM_008229	yes
HELLS I	CGACGAAGTAGAAAGTCAATA	NM_008234	yes
HELLS II	CCGGCTAATCAGGGAGTTAAA	NM_008234	yes
HLTF I	GCCTAATAATCCCTATGATAA	BC057116, NM_009210, NM_144959	yes
HLTF II	GCTATTACACAGGAGTTGTAA	BC057116, NM_009210, NM_144959	yes
Hus1-A	AAGGAACGTTGTTTTGTGTAA	NM_008316.4	yes
Hus1-B	CTGGTTTTAGGTTATAATTTA	NM_008316.4	yes
Hus1-C	AACGAGATTTATTTAGAATTA	NM_008316.4	yes
Lig1	GGCCCGGACATTTGAGAAGAT	NM_001024268, NM_001083188, NM_010715	yes
LIG3 I	GCAAATCGAGATTCACAATAG	NM_010716	yes
LIG3 II	CGAGTCTGTCTGCAAGTAAAT	NM_010716	yes
LIG4 I	GGTGGTTATATAGTACAGAAT	NM_176953	yes
LIG4 II	CGCATAGAAATAGTGCAGAAA	NM_176953	yes
LIG4 III	CCATACAAGGCCGCATAGAAA	NM_176953	yes
Lox	GCCTATATTTGCCTCGACTTA	NM_010728	yes
MDC1	GCCCAATGATGCAGATGAGTA	NM_001010833	yes
MDM2-A	CTGACAGAGAATGATGCTAAA	NM_010786.3	yes
MDM2-B	AAGCTTCAGAGACAAGAACAA	NM_010786.3	yes
MDM2-C	TTGTGTGTTATTAGTTCTTAA	NM_010786.3	yes
MLH1-A	ATGGCTATATATCGAATGCAA	NM_026810.2	yes
MLH1-B	CAGGCATTAGTTTCTCAGTTA	NM_026810.2	yes
MLH1-C	AGGCATTAGTTTCTCAGTTAA	NM_026810.2	yes
Mre11a-A	TCGGATGTTTGTGAATAAAAA	NM_018736.2	yes
Mre11a-B	ATGGAGAAAGATGCAGTTAGA	NM_018736.2	yes
Mre11a-C	TCCGAGAAAGCAGACAGAGAA	NM_018736.2	yes
MSH2	GCCCAGGATGCCATTGTTAAA	NM_000251, NM_008628, NM_031058	yes
MSH2-A	CAGGTTGCAGTTTCATCACTA	NM_008628.2	yes
MSH2-B	CATGCTTGTGTTGAAGTTCAA	NM_008628.2	yes
MSH3	GGCTGGATGATTCTGTAAATA	M80360, NM_010829, XM_001065837	yes

MSH6	GCAGATGAGGCCTTAAGTAAA	NM_010830	yes
MSH6-A	GCAGATGAGGCCTTAAGTAAA	NM_010830	yes
MSH6-B	GAGGTCATTTCTACAGTTCAA	NM_010830	yes
MUS81	CGGGTATACTTAGTGGAAGAA	NM_027877	yes
MUS81-A	TCGGCTGTTAAAAAAAAAAAA	NM_027877.3	yes
MUS81-B	CACGCGTTTCGTGTTTCAAAA	NM_027877.3	yes
MUTYH-A	CAGCTGGTATGACCAAGAGAA	NM_001159581.1	yes
MUTYH-B	TGCCACAGTGATCGACTATTA	NM_001159581.1	yes
MUTYH-C	AGGAGTGTGCTCTCAACACTA	NM_001159581.1	yes
NBS1-A	TAGGTCATTGGTTGTCAGTAA	NM_013752.3	yes
NBS1-B	TGGGATGGACATAGAACTCAA	NM_013752.3	yes
NEIL1	GGAAGCACCCTCCTAAGAGAA	AK013322, NM_028347	yes
NEIL1-A	CAGAAGATCAAGGCCAAACTA	NM_028347.2	yes
NEIL1-B	ACCCTGTGTCTTGCTGGAGTA	NM_028347.2	yes
Nibrin	GCGCTCTTCAAGAAGATGAAA	NM_013752	yes
OGG1-A	CAGCTTGATGATGTCACTTAT	NM_010957.4	yes
OGG1-B	CAGGGACTCAAATTAAAATTA	NM_010957.4	yes
OGG1-C	CAGATCAAGTATGGACACTGA	NM_010957.4	yes
Olfr935	CCATTATTTCTGTGACCTTAT	NM_146746	yes
PALB2	GCTAAGCCACTCTGAATCTAT	AK028653, NM_001081238	yes
PARP1	GGCCATCAAGAATGAAGGAAA	NM_007415, X14206	yes
PIAS1	CCGGATCATTCTAGAGCTTTA	NM_001106829, NM_016166, NM_019663	yes
PIAS1-B	CGGCTGTGTTAGAGTCACAAA	NM_019663.3	yes
PIAS2	GCCCTCTATCTTCAGATGAAT	NM_008602	yes
Pif1	GCAGATGTTCAGATGAAGTAA	NM_172453	yes
Pif1-A	CAAGCCCAAGATAGAAGTTTA	NM_172453.3	yes
Pif1-B	CTCACAGTTCTAGAAGATGAA	NM_172453.3	yes
Plk1 I	CCAGGACCACACCAAACTTAT	NM_011121, NM_017100	yes
Plk1 II	GCAACGGCACCGTGCAGATTA	NM_011121, NM_017100	yes
PMS1 I	GGACATCTGCTGATGACTTTA	NM_153556	yes
PMS1 II	CGGCTTACAGCCTATGACTTA	NM_153556	yes
PMS1 III	GGAGAACTATGGATTTGATAA	NM_153556	yes
PMS1 IV	CCACTTTGTCCTATCATGTAT	NM_153556	yes
PMS2 I	GGTCTTACAGGCGTACTGTAT	NM_008886	yes
PMS2 II	GGAGATCTTGGGTCAGTTTAA	NM_008886	yes
PNKP-A	CAGGACTGTCTGGCTCTCTAA	NM_021549.2	yes
PNKP-B	CAAGTTGGTAATCTTCACCAA	NM_021549.2	yes
PNKP-C	TCAGAAGTGACTCTACCTATA	NM_021549.2	yes
PolB I	GCTAAGAAACTGCCAGGAGTA	NM_011130	yes
PolB II	GGAAGTTTGTAGATGAAGGAA	NM_011130	yes
PolB III	GCTGCAAGGAAGTTTGTAGAT	NM_011130	yes
POLG	GCAGATGTATGCAGTCACAAA	NM_017462	yes
POLG2 I	CCTCCAGAGAAAGGTGCTTAA	NM_001107060, NM_015810	yes
POLG2 II	GCATTTCTTGAGAACTTATTA	NM_001107060, NM_015810	yes
POLH I	CCCAGCTGTACCAACCTCTAA	NM_030715	yes
POLH II	CCAGTTAAACCCAGGCAGTTA	NM_030715	yes
POLI I	GCCAGTTAAAGGATGAGCAAA	NM_011972	yes

POLI II	CCAGGGCATAACCAAATAGAA	NM_011972	yes
POLI III	CGGAAAGGACTGTTCCTATTA	NM_011972	yes
POLK	GCATTTAAAGTGCCAGAAGTA	NM_001107333, NM_198600	yes
POLM I	GGCCTTTAGCAGTTCATACAT	BC056996, NM_017401	yes
POLM II	CCGCATTCCAGCCTAGGCTAA	BC056996, NM_017401	yes
POLN I	GGCTGTGTGATGTTAGATATT	NM_181857, XM_001065133, XM_573636	yes
POLN II	GCAGGAGAACAGTTTCTTATA	NM_181857, XM_001065133, XM_573636	yes
POLQ	GCAGATTTGCTATGGAATTAT	AY135563, NM_001105878, NM_029977	yes
PPM1A I	CCTCTTTATGAGTTCTTACAT	NM_008910	yes
PPM1A II	GCAGAGTAGAAGAAATCATAA	NM_008910	yes
PPM1A III	CCTATTGATGTCTTGGCACTT	NM_008910	yes
PPM1D-A	CAGGGCTAATGTTGAAGAAAA	NM_016910.3	yes
PPM1D-B	AAGGACATTAGAAGAATCCAA	NM_016910.3	yes
PPM1D-C	AGGGAATAAGTTGCTGTAAAA	NM_016910.3	yes
PPP4C	CGATGAGTGCTTACGGAAATA	NM_019674, NM_134359	yes
PRKDC I	GGCAATGCTGTCTACACAATA	NM_011159	yes
PRKDC II	GCCAATGGACTCCTGGAATTA	NM_011159	yes
PRKDC III	CCTGGGAACCTGAAAGAGTAT	NM_011159	yes
RAD1 I	GCAGCACCAATGTTATGAATA	AF073523, NM_001106419, NM_011232	yes
RAD1 II	GCTACTGAAGCCCTCTACAAA	AF073523, NM_001106419, NM_011232	yes
RAD17 I	GCACCAACAATTATGATGAAA	NM_001024778, NM_001044371, NM_011233	yes
RAD17 II	GCATGAACTTGCTGTTCATAA	NM_001024778, NM_001044371, NM_011233	yes
RAD17 III	GCCCTCCACATTAGAAAGTAA	NM_001024778, NM_001044371, NM_011233	yes
Rad18 I	GCCTCTTAGATGAACTGGTAA	NM_001077673, NM_021385	yes
Rad18 II	GCCTCTTAGATGAACTGGTAA	NM_021385	yes
Rad21 I	GGCGGTATATTAGATGACAAA	NM_009009	yes
Rad21 II	GCTTATAATGCCATTACTTTA	NM_009009	yes
Rad23a I	GCAGTTAATTGGTTTGAACTT	AK045224	yes
Rad23a II	GGATTTCCAAGTAGAGTGTAT	AK045224	yes
Rad23a III	GCCACAGAGCTCTTCTGAGAT	AK045224	yes
Rad23a IV	GGCTTCTGTTTACCAACATTA	AK045224	yes
Rad23a V	CCAGTAGGCATGAACCATTAT	AK045224	yes
Rad23b	GCTATGAACGAGAACAAGTAA	NM_001025275, NM_009011	yes
Rad50 I	CGACCATCATTGAATGTCTAA	NM_005732, NM_009012, NM_022246	yes
Rad50 II	CCAGTTATGAAGATAAGCTAT	NM_005732, NM_009012, NM_022246	yes
Rad51 I	GGAAATTTGTTCTGCTGCTAA	NM_011234	yes
Rad51 II	GCACACAGCGTAATAGCCAAA	NM_011234	yes
Rad51 III	GCTTGTTCTCTAAATTACTAA	NM_011234	yes
Rad51AP1 I	CCAGTGATGATGTAGAAGATT	NM_009013	yes
Rad51AP1 II	GCCAAGTCACTGTTAAAGTTA	NM_009013	yes
Rad51B	GGAACATTTCCGAATAAAGTA	NM_009014, NM_133510	yes
Rad51C-A	AAGCACTGTTCTATAATTATA	NM_053269.3	yes
Rad51C-B	CACAGATTAGCTGTTATTTTA	NM_053269.3	yes
Rad51C-C	AAGAGAATGTCTCACAAATAA	NM_053269.3	yes
Rad51D I	CCTCATGTCTTAGTGAATAAA	NM_011235	yes
Rad51D II	CCACATAATGGGAAGAGAGAA	NM_011235	yes
Rad51D III	CCTGCTAGGCTTTCACTGTAT	NM_011235	yes

Rad51D IV	GCCATCCATCTGAGAGTCATA	NM_011235	yes
Rad52 I	GCATTTGTAAAGGTGCAGTTA	NM_011236	yes
Rad52 II	GGAAACTGTATTCTGGACAAA	NM_011236, NM_134424	yes
Rad52B	GCAGTCCTCGTTTCTTATGAA	NM_025654	yes
Rad54l I	GCAGTCAACATGAAGCATTTA	NM_009015	yes
Rad54l II	GCTCTCAACTGCTTAGTTAAA	NM_009015	yes
Rad54l III	GCACTTTAAGACATTGCGAAT	NM_009015	yes
RAD9	CCTCTTACTATCCACTTCGAT	NM_011237	yes
RAD9A-A	TGGGGTCAAGAAGACACACAA	NM_011237.2	yes
RAD9A-B	CTCGATTTTTGTGCTTATGTA	NM_011237.2	yes
RAD9A-C	TTGGTCTGTGTTCTGTATTTA	NM_011237.2	yes
RAD9B	GCTCTTGGAAGCAAACTTTAT	NM_144912	yes
RAD9B	CCATTGCTCTTCTGACATCAA	NM_144912	yes
Raptor I	CGTGGCAAGTTTGTTTAGAAA	NM_028898, XM_001081775, XM_213539	yes
Raptor II	CCTATCCAGATGTTTCTGATT	NM_028898, XM_001081775, XM_213539	yes
RBBP8 I	CCACGGAAACGAGACTTCTAA	AK041384, NM_001081223	yes
RBBP8 II	GGTCAGAAGTGAAGGTCATTA	AK041384, NM_001081223	yes
RBBP8 III	GCCTCCAGTCCTCTAGTAAAT	AK041384, NM_001081223	yes
RECQL I	GGACATTGTCAAGCTAATTAA	NM_023042	yes
RECQL II	GGTAACTCCCAGAAGTCTAAA	NM_023042	yes
RECQL III	CCAGCTCTAAGGAACATGTAA	NM_023042	yes
RECQL5b	GGCTCTAGGAAGCAGTGTTAA	NM_130454	yes
REV3L I	GCCCTTGAACTTACAAGGAAA	NM_002912, NM_011264	yes
REV3L II	GGGTGTAATCGTAATTGATAA	NM_002912, NM_011264	yes
Rmi1	CCTAAAGAAACGGTTGAATAA	NM_028904, XM_977690	yes
RNF168	GCTTCCAGTCCACTGTTGAAA	NM_027355	yes
RNF168-A	CTGGACAAGAATCAAAGGAAA	NM_027355.2	yes
RNF168-B	CAGAAGAAAAATACAAGTAAA	NM_027355.2	yes
RNF8-A	CAGGCTCTAATGGAAGAACTA	NM_021419.1	yes
RNF8-B	AAGGACATGTGAGAGACTCTA	NM_021419.1	yes
RNF8-C	CAGGGTTTGGAGAAAGAGCAA	NM_021419.1	yes
RRM1	GCCCAGAAGCCCAGTTATTAA	NM_009103, XM_001052982, XR_007992	yes
RRM2-A	CTAGAAGAAAGTATACTTCAA	NM_009104.2	yes
RRM2-B	CAGGCTGGTCACTAGAAGAAA	NM_009104.2	yes
RRM2-C	CCCGATGAGAGACATTTTATA	NM_009104.2	yes
RRM2b	GCGCTGGATAGCAGATAGGAA	NM_199476, XM_001061249, XM_235367	yes
RRM2b-A	CAGGACGATCTGATCCTACTA	NM_199476.1	yes
RRM2b-B	CACACACAGAGTACAAATAAA	NM_199476.1	yes
RRM2b-C	CACACACAGAGTACAAATAAA	NM_199476.1	yes
RTEL I	GCCAAGGAGTTCGTACCCTTA	NM_001001882	yes
RTEL II	GGACATCATCTTTATGCCATA	NM_001001882	yes
RTEL III	GCTCACAATGTGGAGAAGATA	NM_001001882	yes
RTEL IV	GGCTTCAGGACTGGAGATTAT	NM_001001882	yes
Securin-A	ACCGAGAAGTCTACTAAGACA	NM_001131054.1	yes
Securin-B	CAGATTGTGAATACTTAAAAA	NM_001131054.1	yes
Securin-C	TTCTGTATGTATGTTGTATTA	NM_001131054.1	yes
SF3B4 I	GGTGTTTACATTGTAAGGATT	NM_153053	yes

SF3B4 II	GCCGACTATGCCATTAAGATT	NM_153053	yes
Slx1-A	TAGGTCAGTTCTCATGAACTA	NM_029420.4	yes
Slx1-B	TTGCAGAGGACTCAAATTCAA	NM_029420.4	yes
Slx1-C	CAGGATGCTGACCTAGAGAAA	NM_029420.4	yes
SLX4-A	AGGGGTTGTTCTTCTGTCAAA	NM_177472.4	yes
SLX4-B	CCAGATGGTTCTAAAACTGAA	NM_177472.4	yes
SMC3 I	GCTCTAGATCAGTTTGTGAAT	NM_007790	yes
SMC3 II	GCTGCTAAATGAAAGAATTAA	NM_007790	yes
SUMO1 I	GCGATAAGAAGGAAGGAGAAT	NM_009460	yes
SUMO1 II	GGCGATAAGAAGGAAGGAGAA	NM_009460	yes
TIPIN I	GGCTTGTTTGAAATACCAGAT	NM_001025287, NM_025372, XM_001075652	yes
TIPIN II	CCTGTATAAGTCTGCCTTTAT	NM_001025287, NM_025372, XM_001075652	yes
Tnik	GGCACGTATGGTCAAGTTTAT	BC050866, XM_001473602, XM_001473621	yes
TOP3A	GGTTACGATTCCATGGGTTAT	NM_009410, XM_001473422	yes
TopBP1 I	GGAATTACAATCAGAAGATTA	NM_176979	yes
TopBP1 II	CGAAGCTGCTTTATTCCATAA	NM_176979	yes
TopBP1 III	GCATCTTCCTGAATCTCTTTA	NM_176979	yes
TP53BP1 I	CCCATGGATATGTCAGTGATA	NM_001106501, NM_013735, XM_001080941	yes
TP53BP1 II	GCCTACAACTGCTGAGAGAAA	NM_001106501, NM_013735, XM_001080941	yes
TP53BP1 III	CCAGTGAAGAATCTTTGCAAT	NM_001106501, NM_013735, XM_001080941	yes
Tssk2	GCTGAGACTTCCAGAGCTAAA	NM_009436	yes
UBE2A-A	CAGGATGTTATTTGTAACAAA	NM_019668.3	yes
UBE2A-B	AAGCGTGTTTCTGCAATAGTA	NM_019668.3	yes
UBE2A-C	TGCGAGATATTCATAGTGTAA	NM_019668.3	yes
UBE2N I	GGGAAGAATATGTTTAGATAT	NM_053928, XM_621074, XM_910030	yes
UBE2N II	GGCTATATGCCATGAACAATA	NM_053928, XM_621074, XM_910030	yes
WRN I	CCATCAACTCAGATATGTATA	NM_011721, XM_001059940, XM_001471964	yes
WRN II	CGCAACATTCCTCTAATCAAA	NM_011721, XM_001059940, XM_001471964	yes
Xab2 I	CCACAGATCGTTATTAACTAT	NM_026156	yes
Xab2 II	CCCAATGAAGTCCGACTGGAA	NM_026156	yes
Xab2 III	CCTGTGCAGAACCGTGTATAT	NM_026156	yes
XLF	CCACCCTCTCAGAGCCTGAAT	AK006481, NM_029342	yes
XLF-A	TAGGTACATGAAGCAAGTTTA	NM_029342.4	yes
XLF-B	CAGATGGAAGAGCTAGAGCAA	NM_029342.4	yes
Xpa	CGATATGAAGCTCTACCTAAA	NM_011728	yes
Xpb	GCAGTGGAAAGCCCAGTTTAA	NM_001031644, NM_133658	yes
Xpc	GCTGCCAAGGTGTCATACAAA	NM_009531	yes
Xpd I	CCAGAGATTGAGAAGGTCATA	NM_007949	yes
Xpd II	GGTAGCAATCAGCTCCAAATT	NM_007949	yes
Xpe-A	CAGACAAGAGTTCTCATGCTA	AB026432.1	yes
Xpe-B	AACCAGTTCACTAGAGACCTA	AB026432.1	yes
Xpe-C	CCGAGACAATAAAGAGCTCAA	AB026432.1	yes
Xpf I	TCCCTGGCACAGGATGGTGAA	NM_005236	yes
Xpf II	CGGCCCGAAGAGAACTACAAA	NM_005236	yes
Xpf III	GCGTGAATCATTTGATTTAAA	NM_005236	yes
Xpg I	GCGAACACTGTTTGAAGCAAT	NM_011729	yes
Xpg III	GCCCGGCATGTCTATAAGAAT	NM_011729	yes

Xpg-A	CCAACGAATGGCAAGATATTA	NM_011729	yes
Xpg-B	CCGCGAGTCACCGATGTGATA	NM_011729	no
XRCC1-A	CCCGGTGGATCTACAGTTGTA	NM_009532.4	yes
XRCC1-B	CCGGTGGATCTACAGTTGTAA	NM_009532.4	yes
XRCC1-C	TTGAGTCTAGAAGACAGGAAA	NM_009532.4	yes
XRCC2	GGAATTATGCTGCTTTAATAA	NM_001109215, NM_020570	yes
XRCC3 I	GCTGTCTACATCTGCACAGAA	NM_028875	yes
XRCC3 II	GCTGTCTAGGCTAAGTGGCTT	NM_028875	yes
XRCC4 I	GGCACATATATCATATTTGTT	NM_028012, XM_001471743	yes
XRCC4 II	GCCCAGAAGATCTCTTTGATT	NM_028012, XM_001471743	yes
XRCC5 I	GGACCAACTGGACGTTATAAT	NM_009533	yes
XRCC5 II	GGAGTTACTCTGATCACCAAA	NM_009533	yes
XRCC6	CGTCAGATTGTGCTGGAGAAA	NM_010247, NM_139080, XM_001067309	yes
